# Supplementary material for: Starch-mediated colloidal chemistry for highly reversible zinc-based polyiodide redox flow batteries
Source: Nat Commun. 2024 May 7;15:3841. doi: 10.1038/s41467-024-48263-8 (PMC11076626; doi:10.1038/s41467-024-48263-8)
Supplement: Supplementary file 1 — Supplementary Information [file 41467_2024_48263_MOESM1_ESM.pdf]

Supporting Information

Starch-mediated Colloidal Chemistry for Highly Reversible Zinc-based Polyiodide Redox Flow Batteries

Zhiquan Wei[1], Zhaodong Huang[1,2], Guojin Liang[3]*, Yiqiao Wang[1], Shixun Wang[1], Yihan Yang[4], Tao Hu[5], Chunyi Zhi[1, 2, 4]*

[1] Department of Materials Science and Engineering, City University of Hong Kong, Hong Kong, 999077, China.

[2] Hong Kong Center for Cerebro-Cardiovascular Health Engineering (COCHE), Hong Kong, 999077, China.

[3] Faculty of Materials Science and Energy Engineering/Institute of Technology for Carbon Neutrality, Shenzhen Institute of Advanced Technology, Chinese Academy of Sciences (CAS) Shenzhen, Guangdong, 518055, China.

[4] Songshan Lake Materials Laboratory, Dongguan, Guangdong, 523808, China.

[5] School of Materials Science and Engineering, Anhui University, Hefei, 230601, China.

* Corresponding author:

Email: gj.liang@siat.ac.cn (G. Liang); cy.zhi@cityu.edu.hk (C. Zhi)

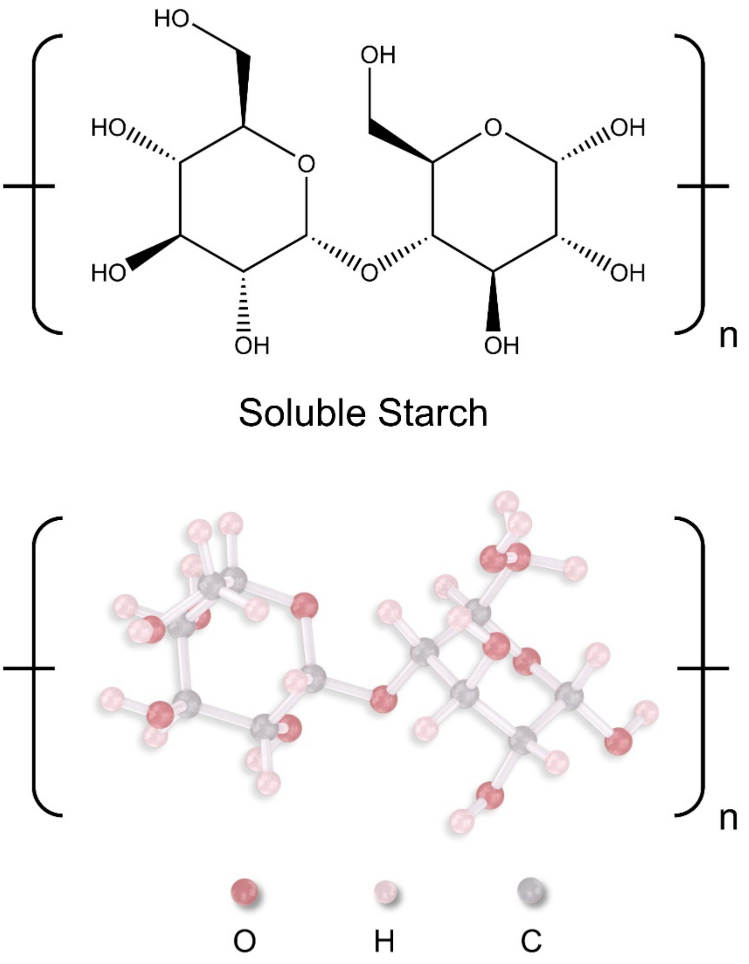

Supplementary Fig. 1 | Structure of starch. The chemical and molecular structure of starch.

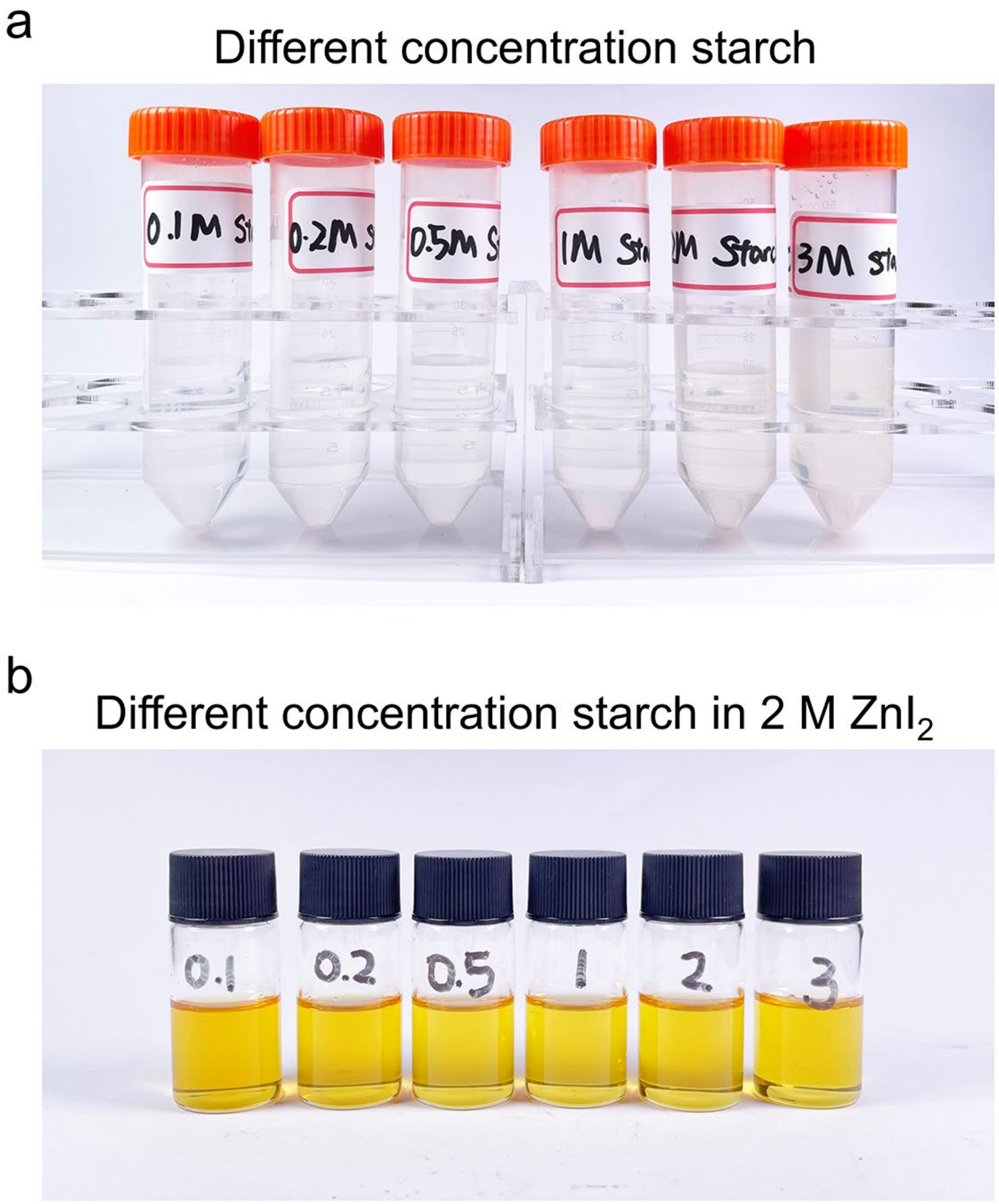

Supplementary Fig. 2 | Starch-based photographs. a Photographs of different concentrations of starch and **b** different concentration of starch in 2 M ZnI_2.

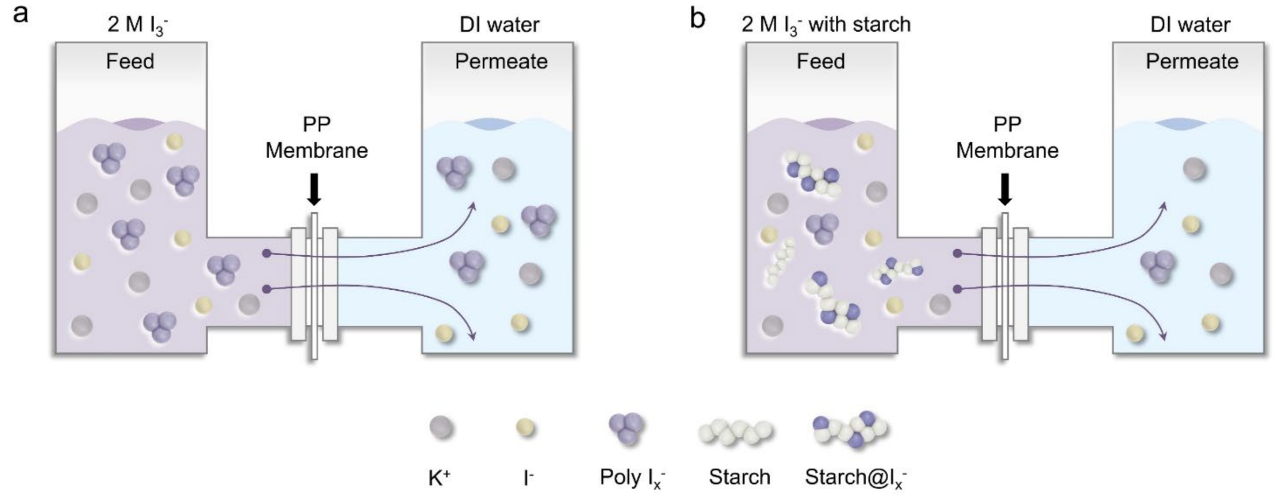

Supplementary Fig. 3 | Ionic permeation measurements. a Two-compartment H-cell testing configuration of 2 M KI_x permeation using PP membrane. **b** Two-compartment H-cell testing configuration of 2 M KI_x permeation with different concentration starch. Note that emphasizing the inhibition of polyiodides cross-over by the colloidal starch, using single salt dialysis (DI water as the permeate side) represented a harsh testing condition, creating a significant osmotic pressure and ionic strength differential environment.

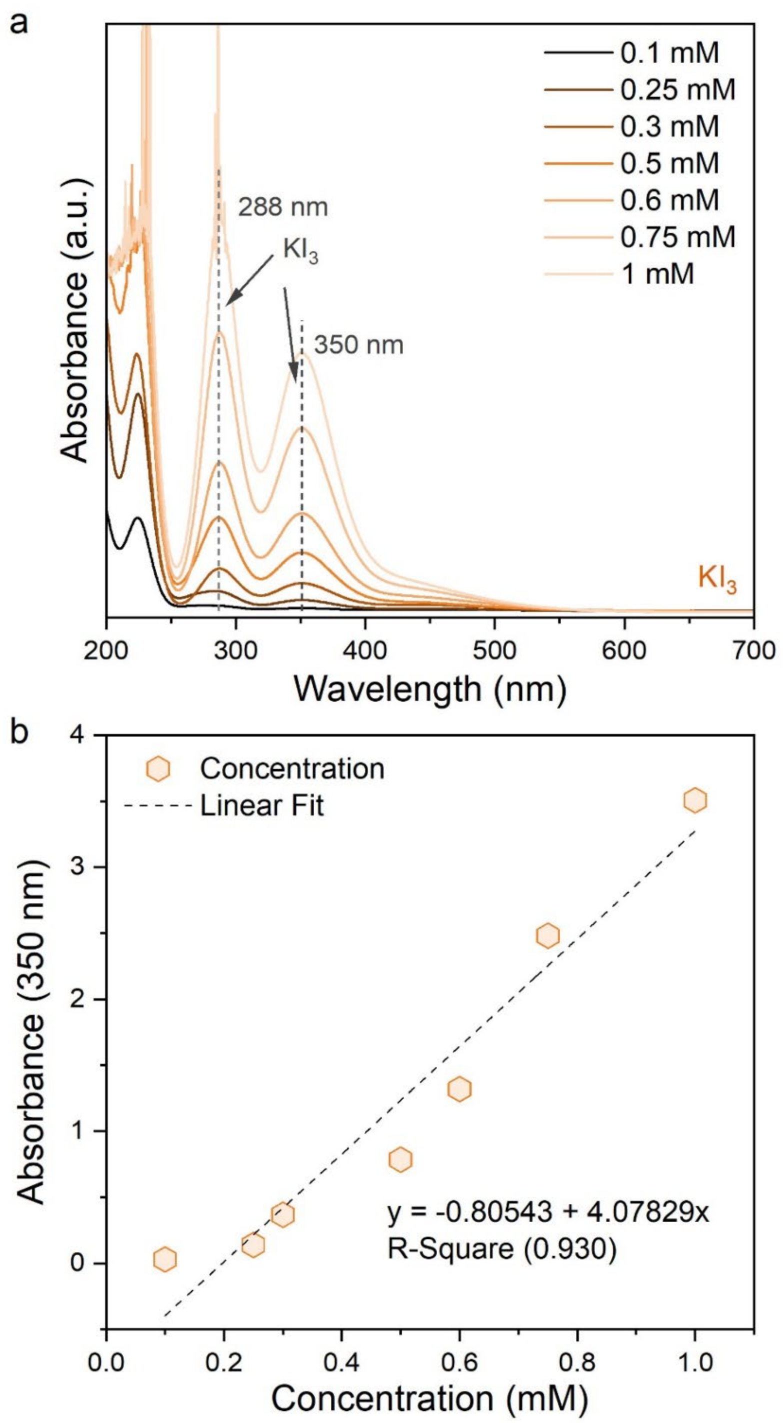

Supplementary Fig. 4 | Standard UV-visible spectra of aqueous KI_3 electrolytes. a UV-visible spectra of KI_3 under multiple concentrations between 0.1 mM to 1 mM. **b** Beer's law plot for I_3 multiple concentrations between 0.1 mM to 1 mM.

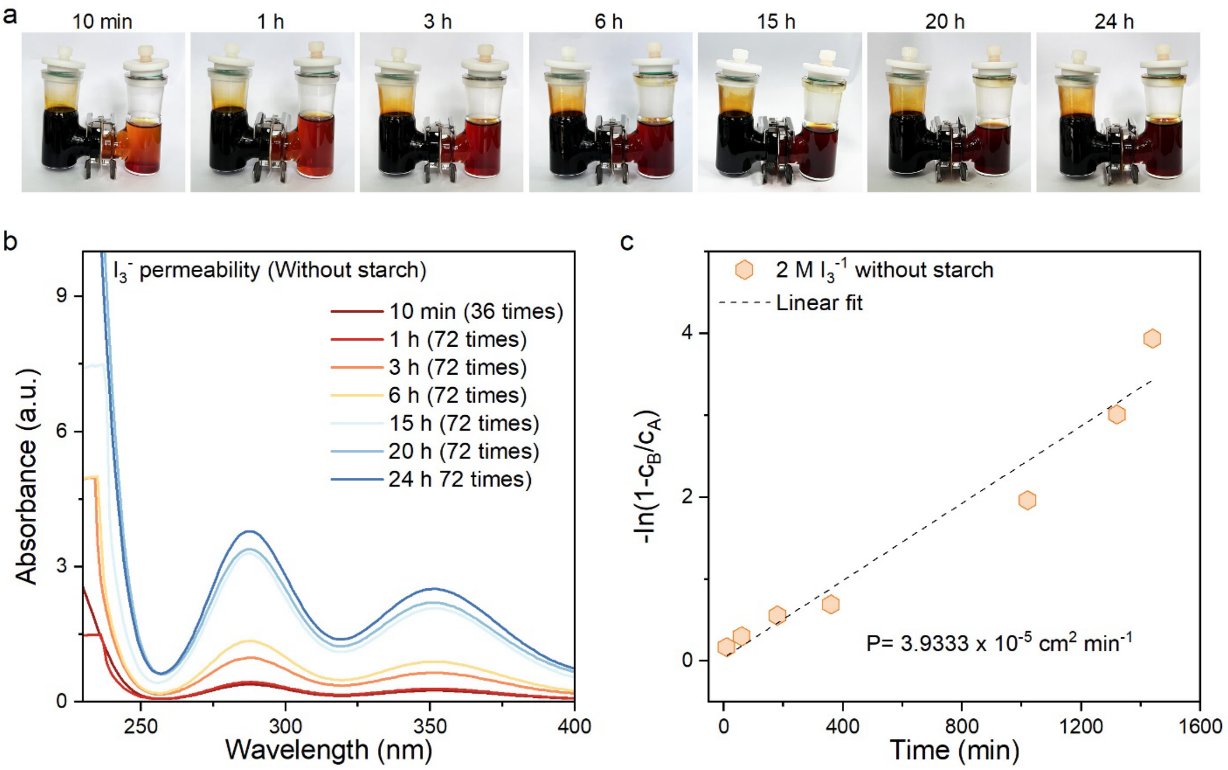

Supplementary Fig. 5 | KI_3 permeation measurements without starch. **a** Photographs of the KI_3 permeate solutions through PP membranes under blank 2 M KI_3 electrolytes. **b** UV-vis of the KI_3 permeated side. (c) $-\ln(1-c_B/c_A)$ *vs*. permeation time for the determination of permeability of KI_3 through PP membranes under blank 2 M KI_3. The fits in the $-\ln(1-c_B/c_A)$ vs. t (time) plots in **c** were obtained by linear fitting.

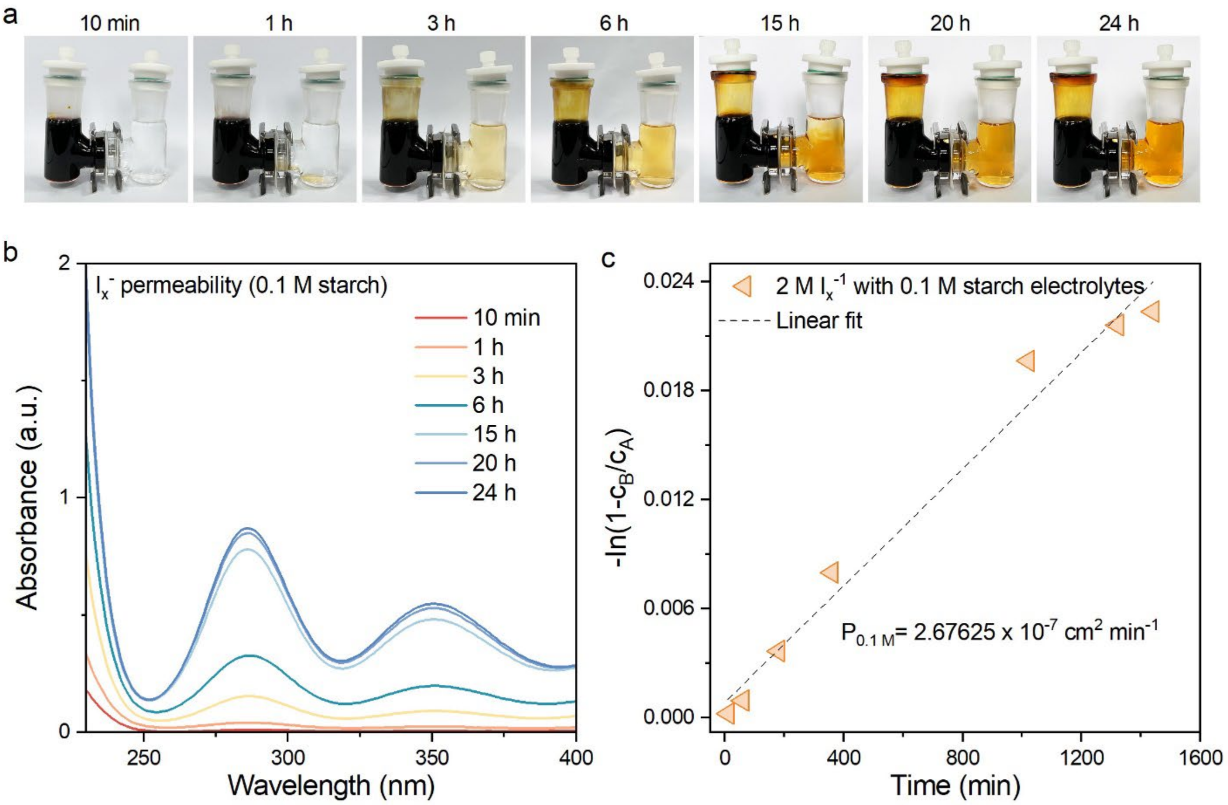

Supplementary Fig. 6 | KI_3 permeation measurements with 0.1 M starch. a Photographs of the KI_x permeate solutions through PP membranes under 2 M KI_x with 0.1 M starch electrolytes. **b** UV-vis of the KI_x permeated side. (c) -ln(1-c_B/c_A) *vs*. permeation time for the determination of permeability of KI_x through PP membranes under 2 M KI_x with 0.1M starch. The fits in the -ln(1-c_B/c_A) vs. t (time) plots in **c** were obtained by linear fitting.

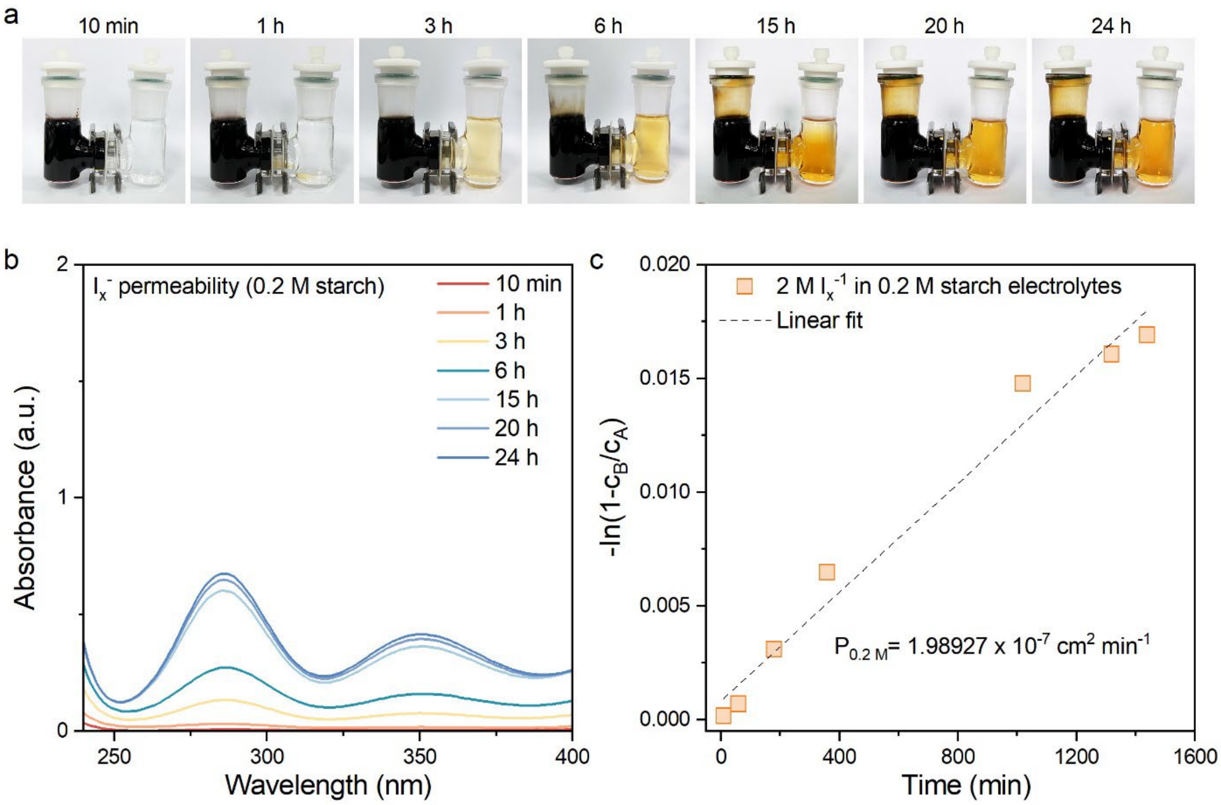

Supplementary Fig. 7 | KI_3 permeation measurements with 0.2 M starch. a Photographs of the KI_x permeate solutions through PP membranes under 2 M KI_x with 0.2 M starch electrolytes. **b** UV-vis of the KI_x permeated side. (c) -ln(1-c_B/c_A) *vs*. permeation time for the determination of permeability of KI_x through PP membranes under 2 M KI_x with 0.2 M starch. The fits in the -ln(1-c_B/c_A) vs. t (time) plots in **c** were obtained by linear fitting.

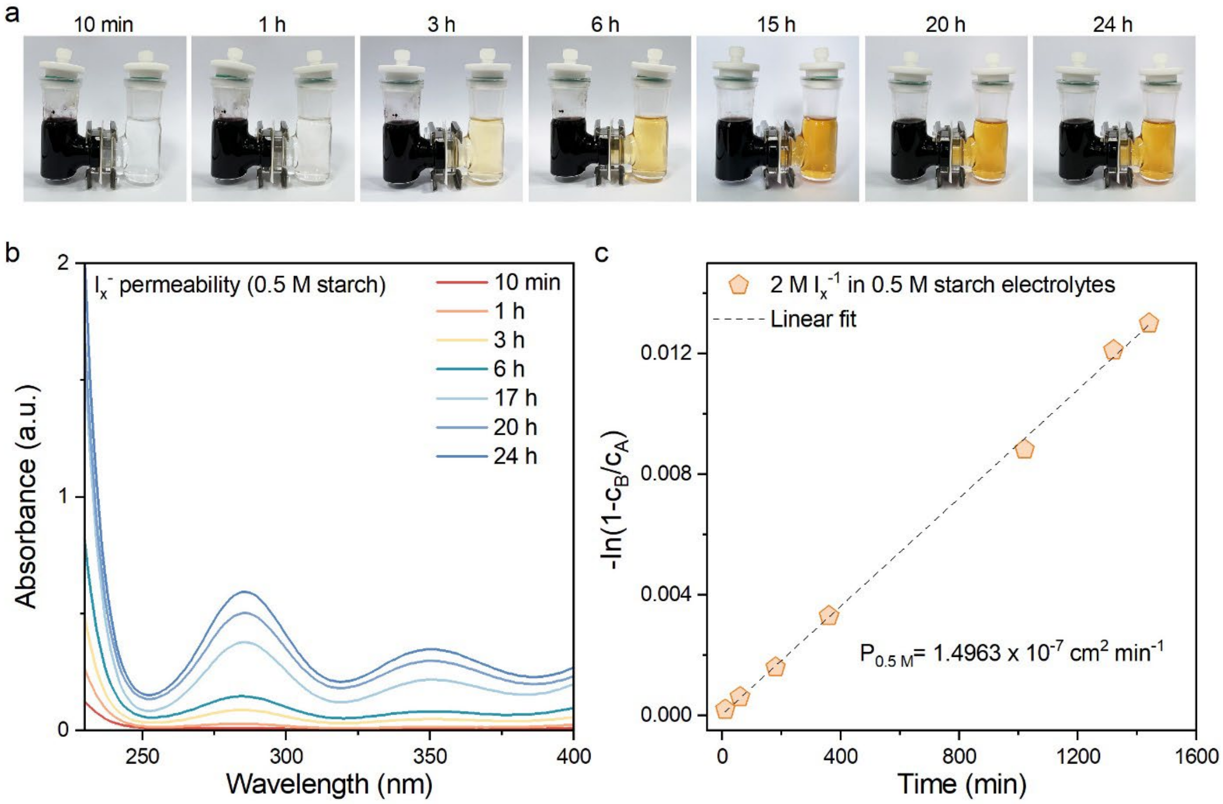

Supplementary Fig. 8 | KI_3 permeation measurements with 0.5 M starch. a Photographs of the KI_x permeate solutions through PP membranes under 2 M KI_x with 0.5 M starch electrolytes. **b** UV-vis of the KI_x permeated side. (c) $-\ln(1-c_B/c_A)$ *vs*. permeation time for the determination of permeability of KI_x through PP membranes under 2 M KI_x with 0.5 M starch. The fits in the $-\ln(1-c_B/c_A)$ vs. t (time) plots in **c** were obtained by linear fitting.

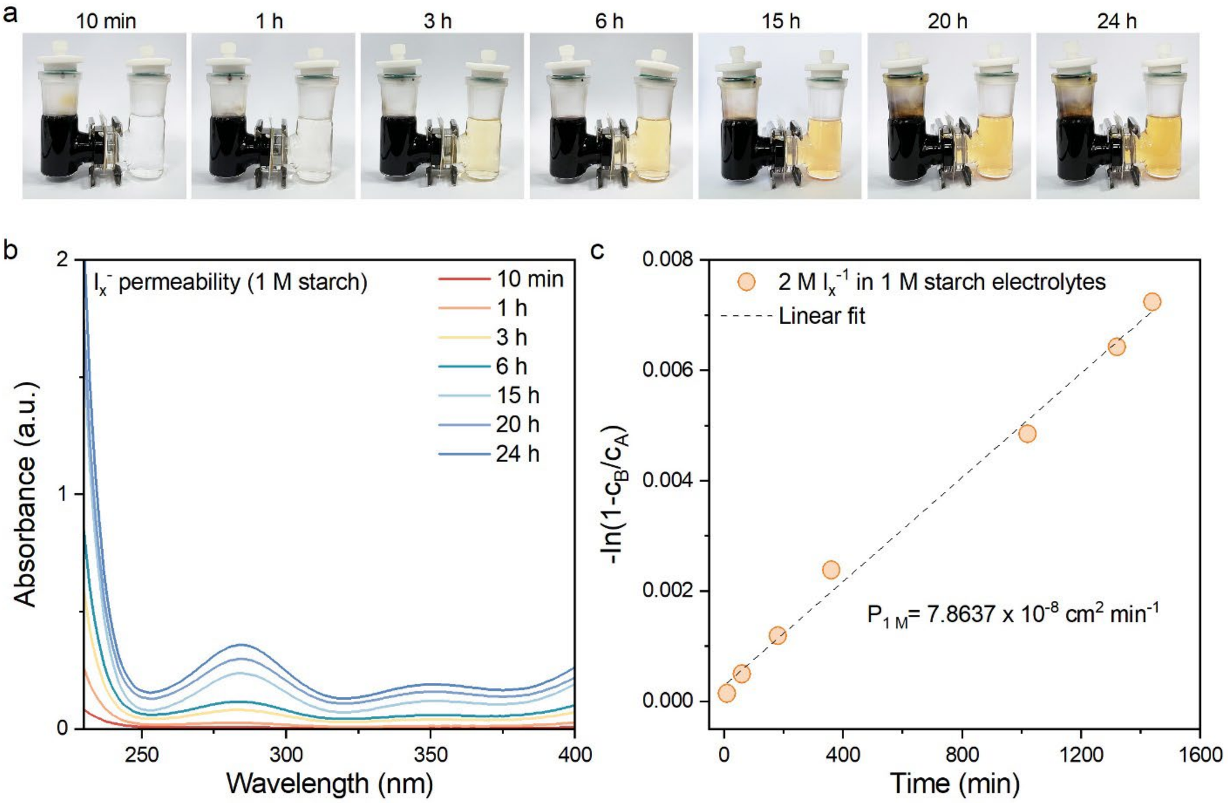

Supplementary Fig. 9 | KI_3 permeation measurements with 1 M starch. a Photographs of the KI_x permeate solutions through PP membranes under 2 M KI_x with 1 M starch electrolytes. **b** UV-vis of the KI_x permeated side. (c) $-\ln(1-c_B/c_A)$ *vs*. permeation time for the determination of permeability of KI_x through PP membranes under 2 M KI_x with 1 M starch. The fits in the $-\ln(1-c_B/c_A)$ vs. t (time) plots in **c** were obtained by linear fitting.

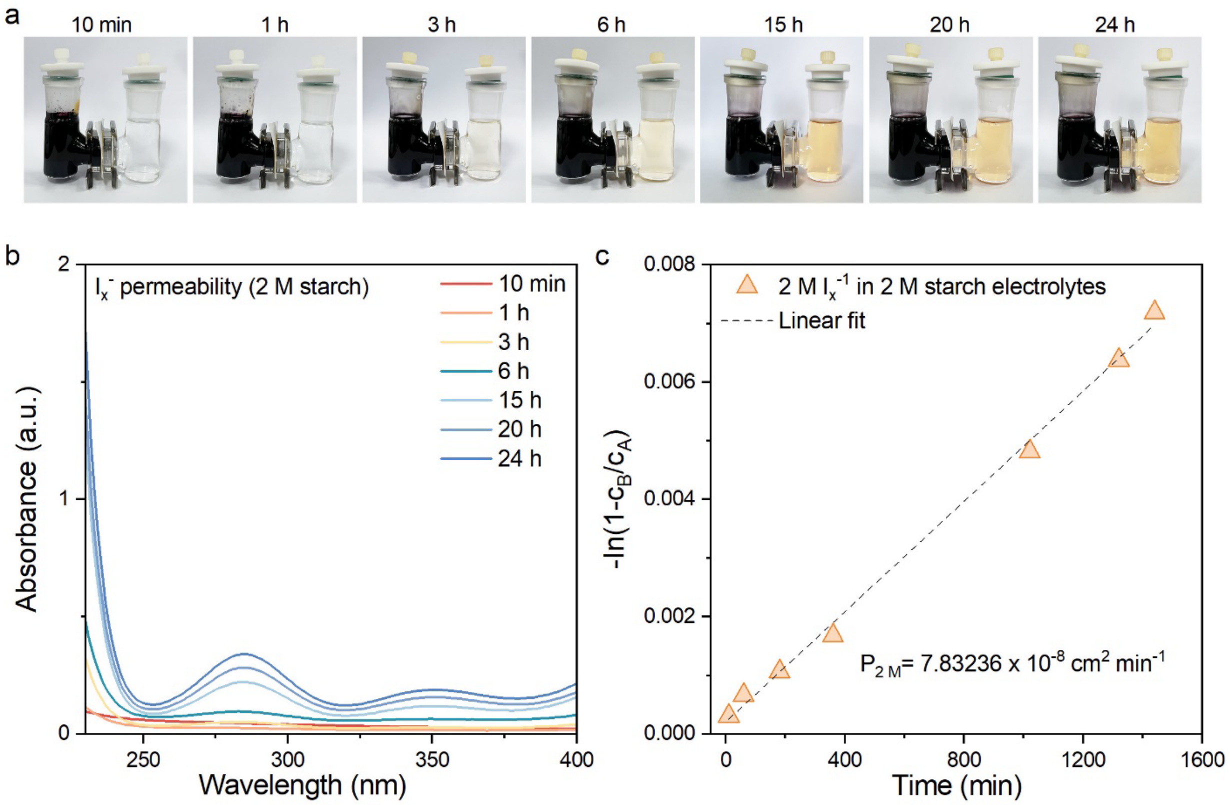

Supplementary Fig. 10 | KI_3 permeation measurements with 2 M starch. a Photographs of the KI_x permeate solutions through PP membranes under 2 M KI_x with 2 M starch electrolytes. **b** UV-vis of the KI_x permeated side. (c) $-ln(1-c_B/c_A)$ *vs*. permeation time for the determination of permeability of KI_x through PP membranes under 2 M KI_x with 2 M starch. The fits in the $-ln(1-c_B/c_A)$ vs. t (time) plots in **c** were obtained by linear fitting.

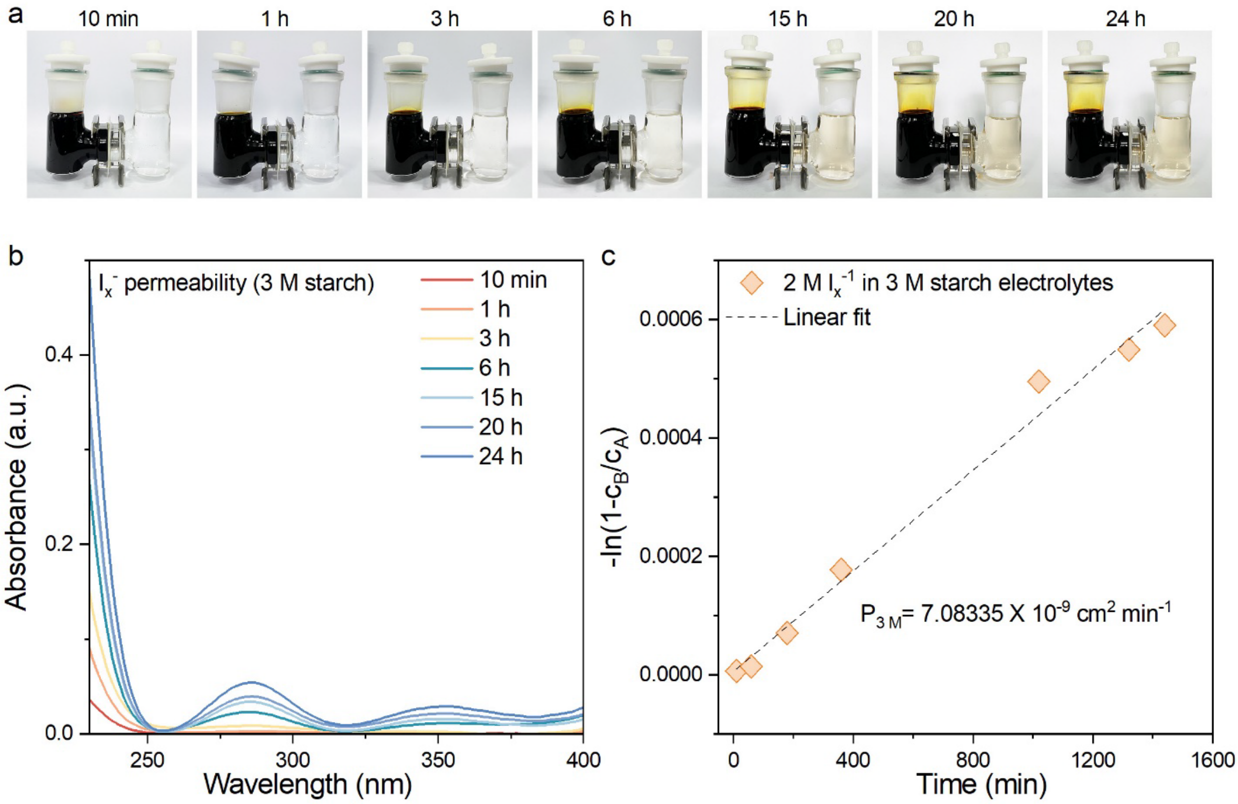

Supplementary Fig. 11 | KI_3 permeation measurements with 3 M starch. a Photographs of the KI_x permeate solutions through PP membranes under 2 M KI_x with 3 M starch electrolytes. **b** UV-vis of the KI_x permeated side. (c) $-\ln(1-c_B/c_A)$ *vs*. permeation time for the determination of permeability of KI_x through PP membranes under 2 M KI_x with 3 M starch. The fits in the $-\ln(1-c_B/c_A)$ vs. t (time) plots in **c** were obtained by linear fitting.

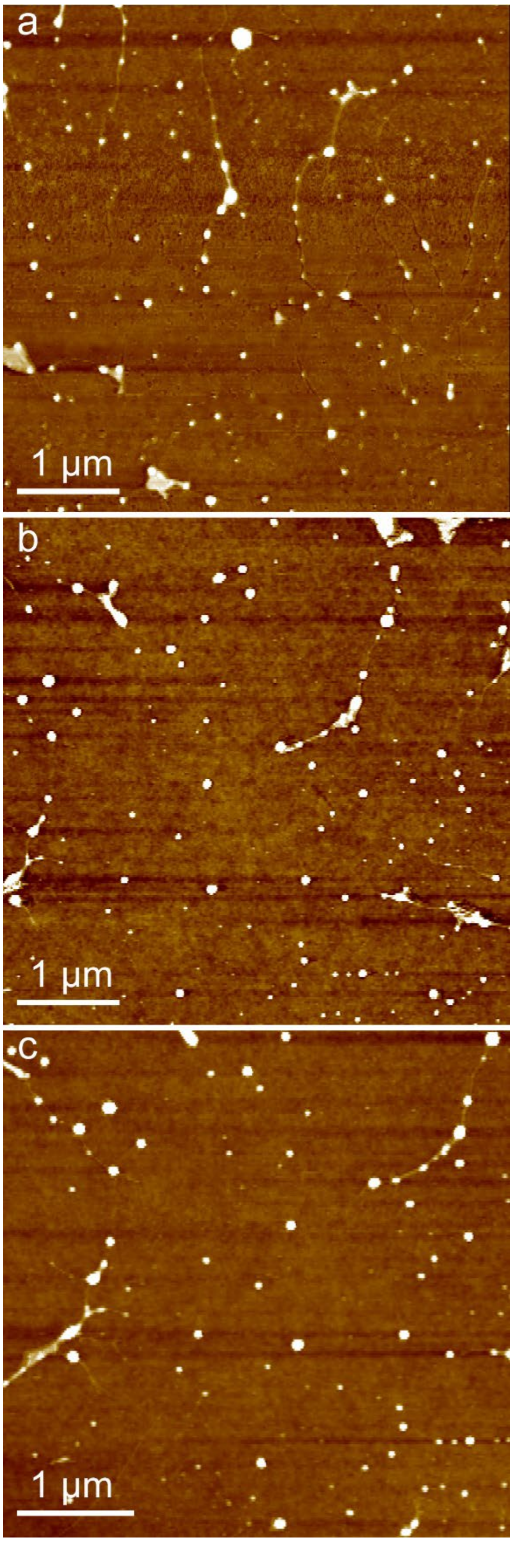

Supplementary Fig. 12 | Characterization of starch. a - c AFM images of 1 M starch solution.

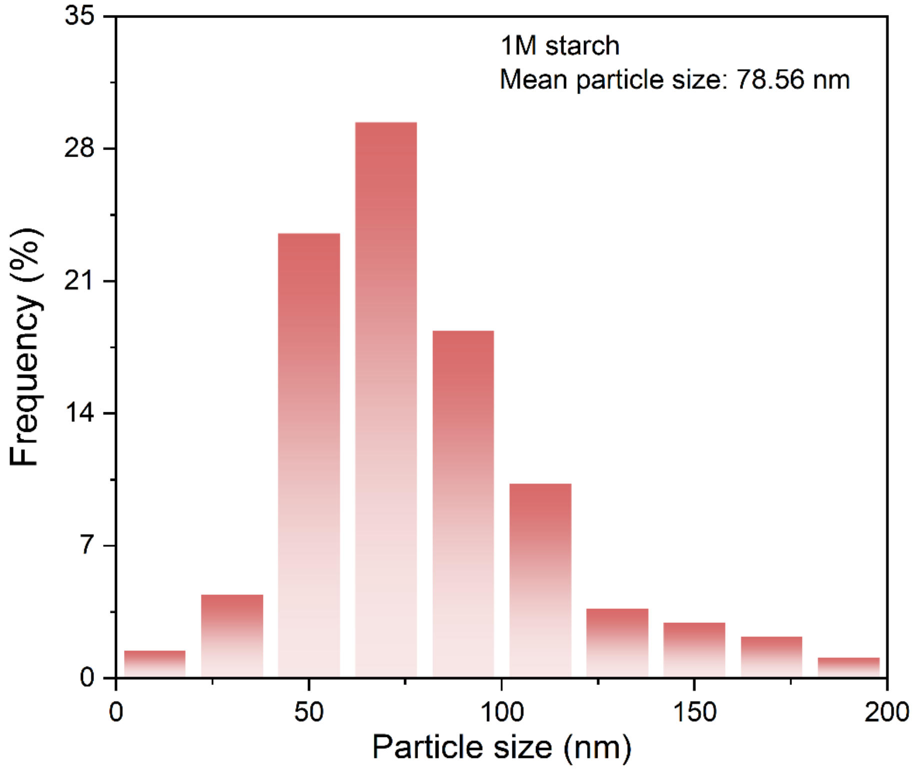

Supplementary Fig. 13 | Size of starch nanoparticles. Particle size distribution of 1 M starch estimated by AFM images.

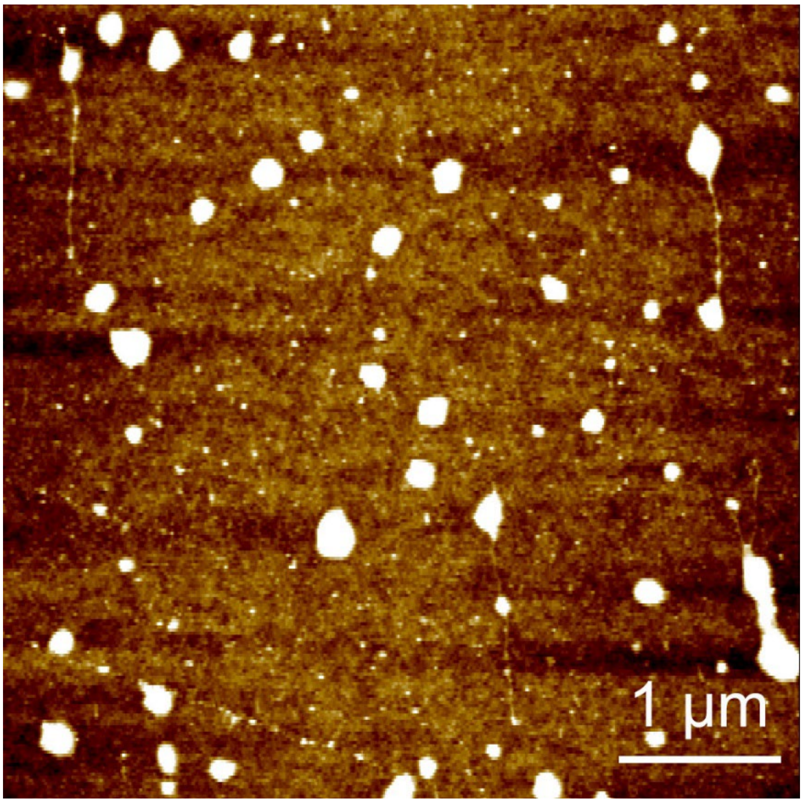

Supplementary Fig. 14 | Characterization of polyiodides-starch complexes. AFM images of 1 M starch solution interacted with I_x^- in 50% SOC.

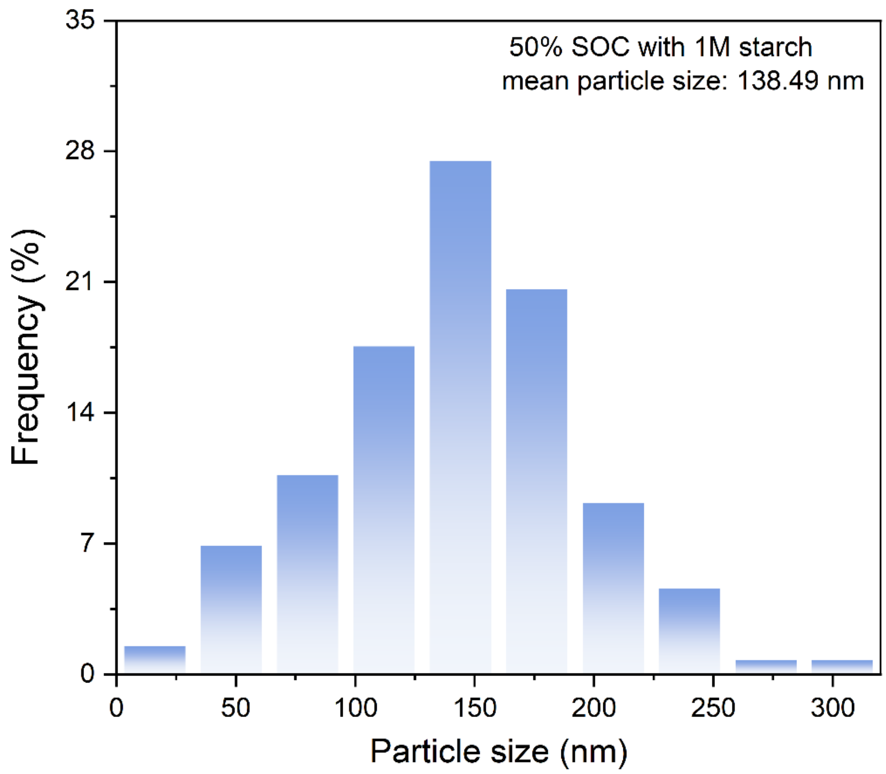

Supplementary Fig. 15 | Size of polyiodides-starch complexes. Particle size distribution of 1 M starch with I_x^- in 50% SOC estimated by AFM images.

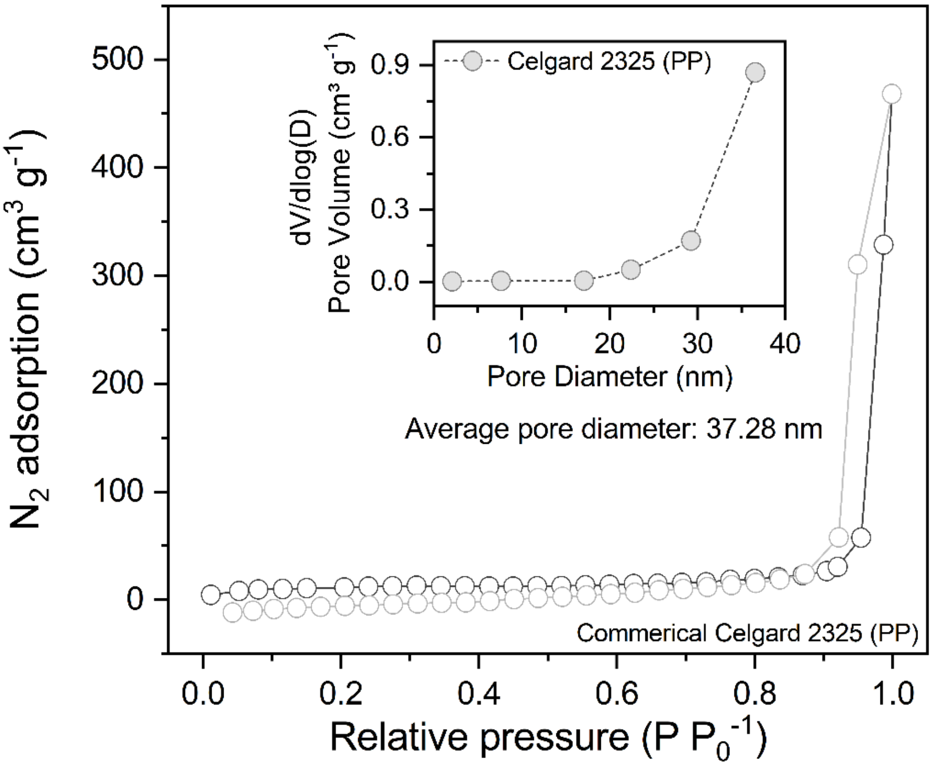

Supplementary Fig. 16 | Characterization of PP membrane. Nitrogen adsorption/desorption curves of PP membrane (the inset: Pore size distribution).

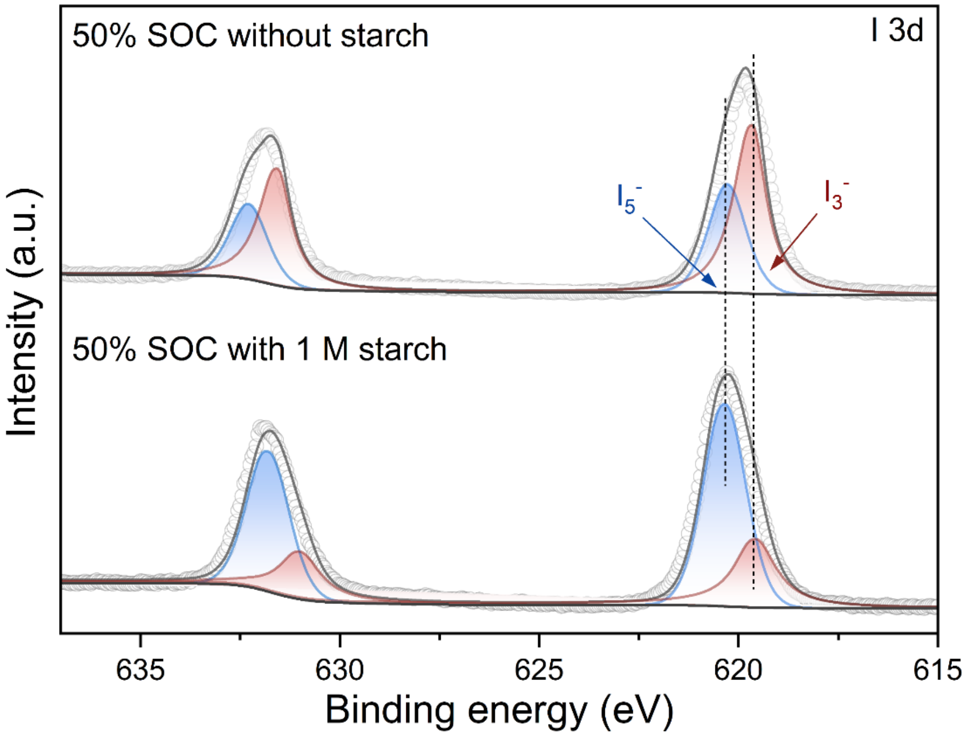

Supplementary Fig. 17 | XPS results of different electrolytes. *I 3d* XPS depth profiles of I_3^- and I_5^- species with/without starch under 50% SOC.

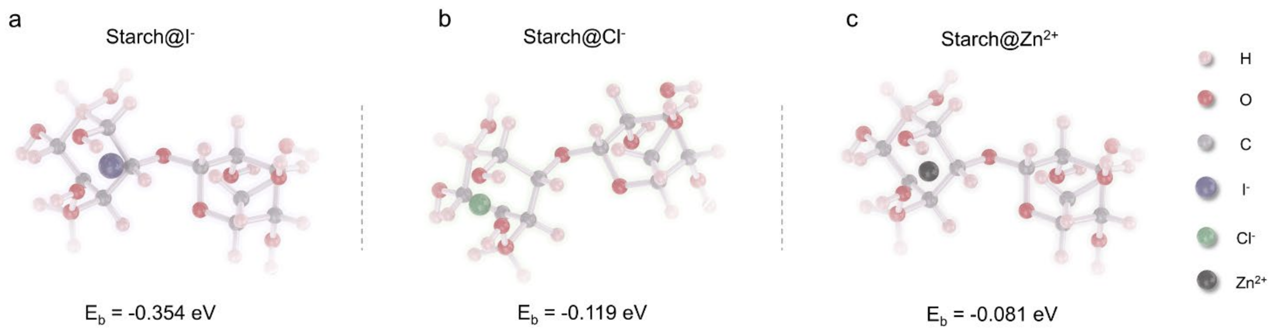

Supplementary Fig. 18 | DFT calculation. The evolution of bonding energy of **a** I^-, **b** Cl^- and **c** Zn^{2+} interacting with the soluble starch.

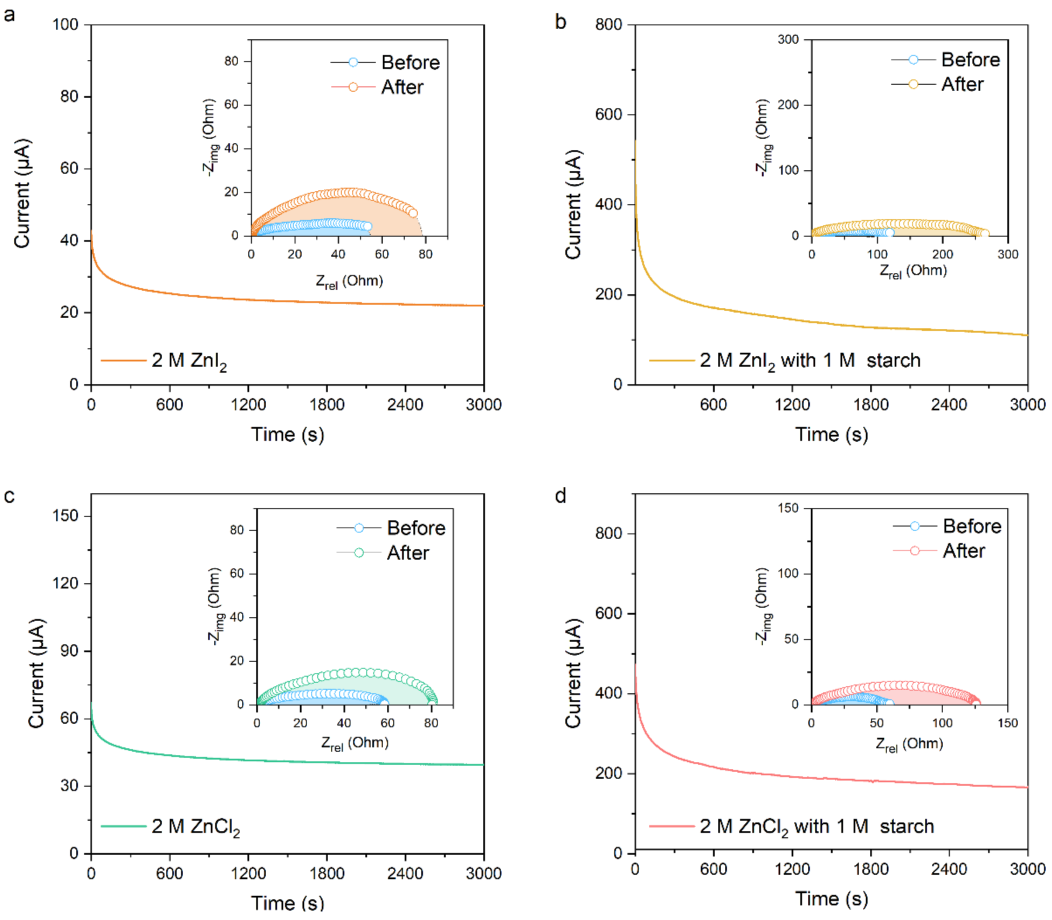

Supplementary Fig. 19 | Ionic transference behaviors in different electrolytes. Chrono-amperometry curves of Zn||Zn symmetrical cell with a static potential of 10 mV in **a** blank 2 M ZnI_2, **b** 2 M ZnI_2 with starch, **c** blank 2 M $ZnCl_2$ and **d** 2 M $ZnCl_2$ with starch. The corresponding EIS plots before and after polarization are shown in the inset.

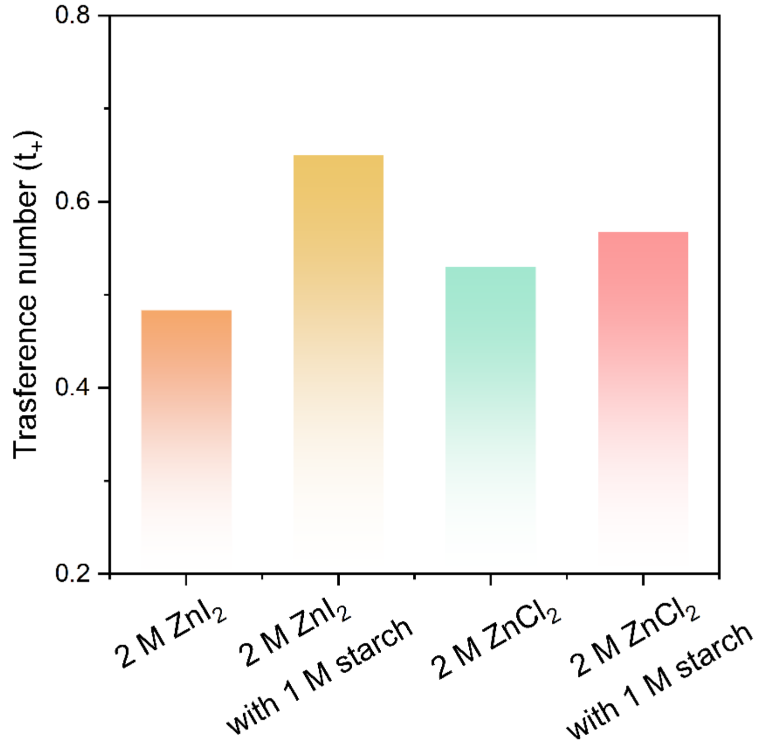

Supplementary Fig. 20 | The comparison of transference number in different electrolytes. Transference number of blank 2 M ZnI_2, 2 M ZnI_2 with 1 M starch, blank 2 M $ZnCl_2$ and 2 M $ZnCl_2$ with 1 M starch.[1]

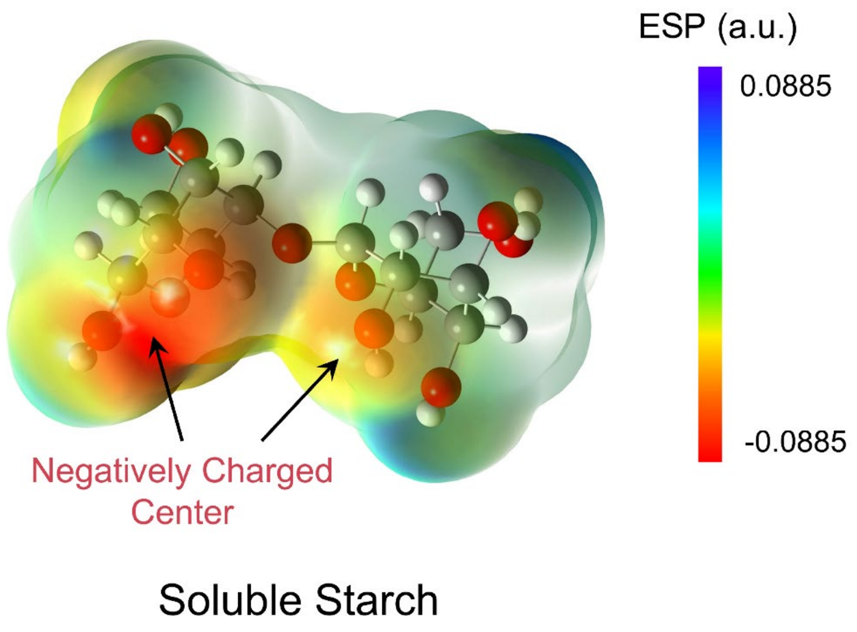

Supplementary Fig. 21 | The electrostatic potential (ESP)-mapping of starch molecules. Surface local minima of ESP are represented as red spheres, and the corresponding ESP values are marked out by numbers. Note that the interaction between starch and iodine relates to the electron donation properties of hydroxyl functional groups. Hydroxyl groups (-OH) are electron-donating, meaning they can donate electrons to other atoms through covalent or hydrogen bonds. In an iodine-starch complex, iodine atoms form a complex with the hydroxyl groups of the starch molecule, forming strong chemical interactions between the oxygen atom of the hydroxyl group and the iodine atom to ensure the stability of the complex.[2-4]

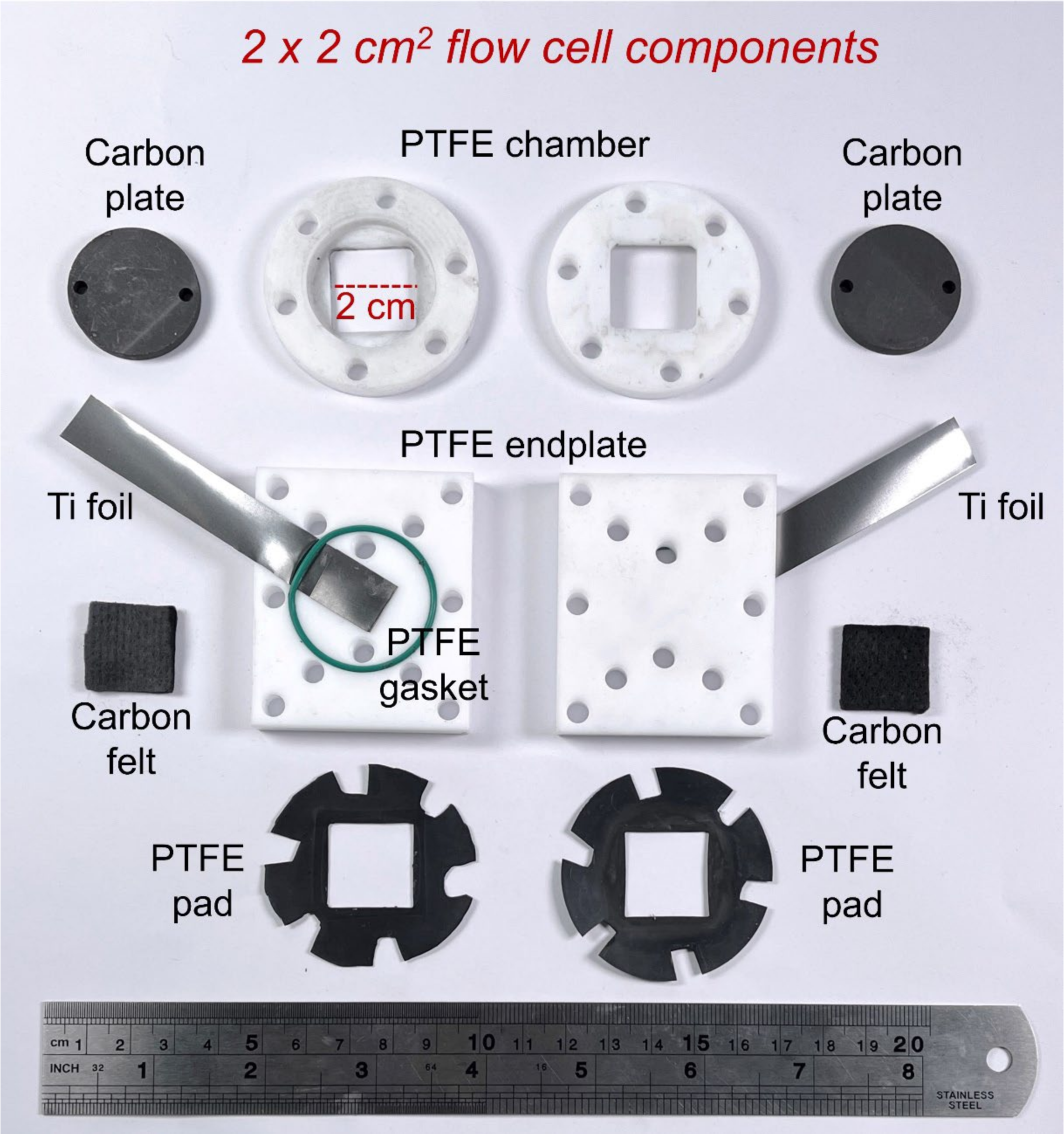

Supplementary Fig. 22 | Digital graphs. Photographs of the cell components of 2×2 cm^2 cell for flow cell tests in **Fig. 3 & Fig. 4**.

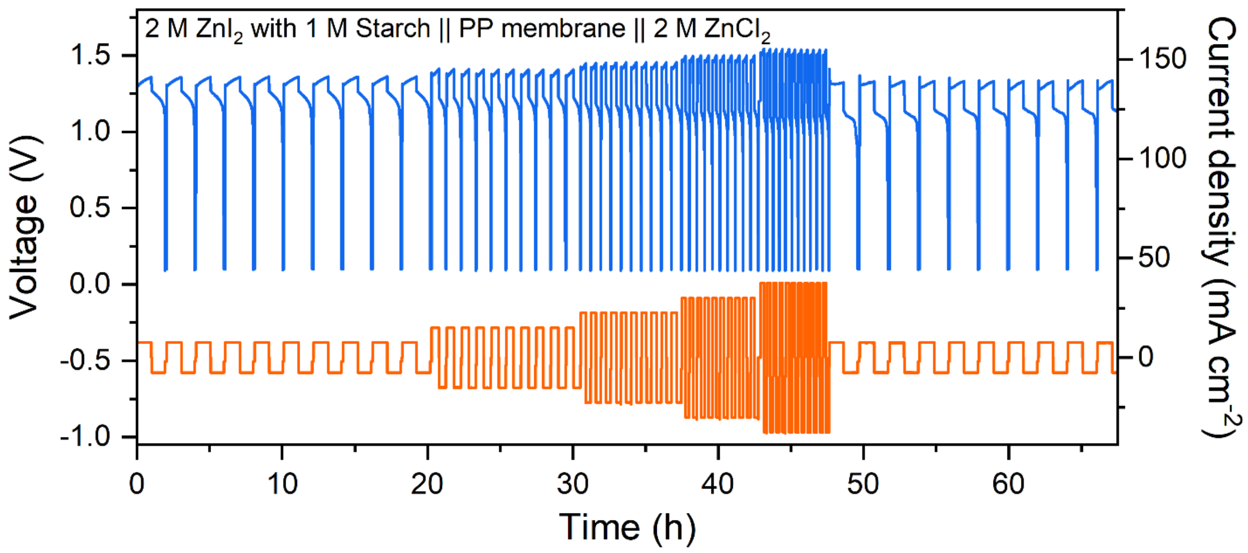

Supplementary Fig. 23 | Power capability of Zn-IS FBs. Galvanostatic cycling of Zn-IS FBs (2 ml of 2 M ZnI_2 with 1 M starch || PP membrane || 8 ml of 2 M $ZnCl_2$, 4 cm^2 membrane area) under 7.5, 15, 22.5, 30, 37.5 and 7.5 mA cm^2 at room temperature. Note that considering critical performance metrics encompassing energy efficiency and cycle life, as shown in **Fig. 2b**, we selected 1 M starch and 2 M ZnI_2 as research objects in this work. It is worth noting that the energy density of 2 M ZnI_2 posolyte at 50% SOC is also competitive compared with other systems. On the other hand, the free movement of the ions and the H_2O molecules across the porous hydrophilic PP membrane (the pore diameter of 37.28 nm in **Fig. 2c**), which could avoid the osmotic pressure of electrolytes to suppress the water migration.

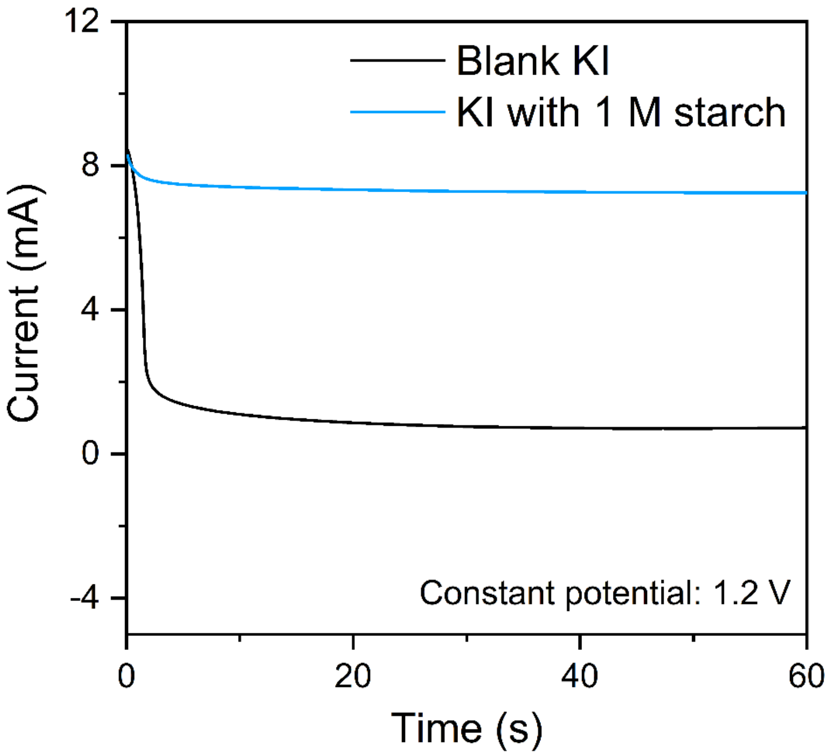

Supplementary Fig. 24 | electrochemical behaviors in different electrolytes. Chronoamperograms in 0.1 M KI and 0.1 M KI with 1 M starch. Nota that to explore the effect of starch on the redox reaction of iodine, we conducted a chronoamperometry (CA) test under the solutions of KI with/without starch at a constant potential of 1.2 V (**Supplementary Fig. 24**). For blank KI solutions, it is observed that the current experiences a sharp decrease in the first two seconds, followed by a steady-state value of 0.74 mA. The decline can be ascribed to the formation of solid I_2 that covers the electrode to passivate the reaction of the active material at the initial reaction process. For KI electrolytes with starch, the current showed a much larger value of 7.29 mA and remained constant during the CA test, which can be demonstrated that starch could rapidly interact with products (I_2/I_3^-), continuously uncovering the fresh surface of the electrode, which favors the redox reaction.

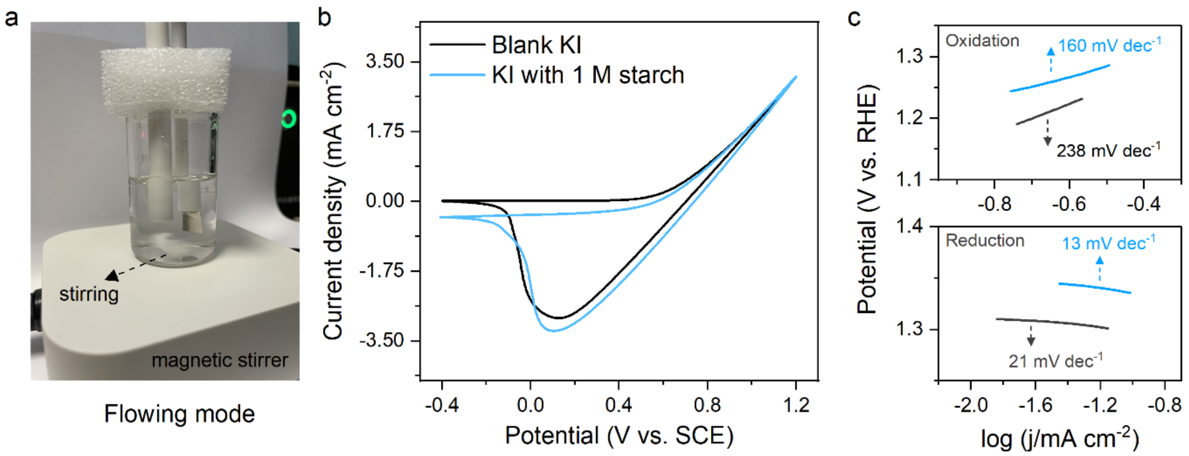

Supplementary Fig. 25 | electrochemical process of iodine-based electrode in different electrolytes. a The photograph of the reactor under flowing-mode conditions. Cyclic voltammetry of redox reactions in **b** 0.1 M KI without and with 1 M starch under flowing-mode conditions. **c** The corresponding Tafel plots in different electrolytes. To simulate a real flow battery environment, stirring was applied within the reactor to ensure the thorough dispersion of the oxidized I_3^- into the electrolyte (**Supplementary Fig. 25a**). The cyclic voltammetry (CV) curve of I_3^-/I^- redox reactions in potassium iodide (KI) electrolytes with/without starch in the potential range of -0.4 to 1.2 V (vs. SCE) was tested by the three-electrode mode. Due to an excess of iodide ions, the oxidation reaction continued, which was unable to show the distinct oxidation peak (**Supplementary Fig. 25b**). Moreover, the Tafel plots were calculated by corresponding CV curves. As shown in **Supplementary Fig. 25c**, the starch-electrode was characterized by lower Tafel slopes for both oxidation (160 mV dec^{-1}) and reduction (13 mV dec^{-1}) steps than those of blank electrolytes-based electrode (oxidation: 238 mV dec^{-1}, reduction: 160 mV dec^{-1}), proving the significantly enhanced reaction kinetics of iodide species in both charging and discharging processes of the Zn-I FBs. Those results can be attributed to the blocked electrode by passivating solid I_2 in blank electrolytes while maintaining the clean surface of the electrode with starch.

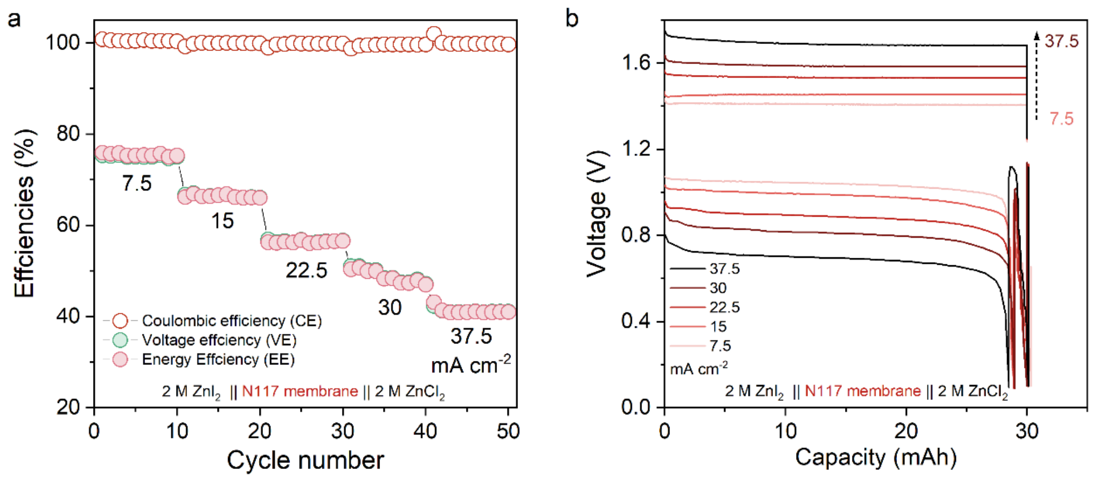

Supplementary Fig. 26 | Rate performance of Zn-I FBs using N117 membrane. a CE, VE, EE and **b** voltage profiles of the Zn-I FBs using N117 membrane without starch under 7.5, 15, 22.5, 30, and 37.5 mA cm^2.

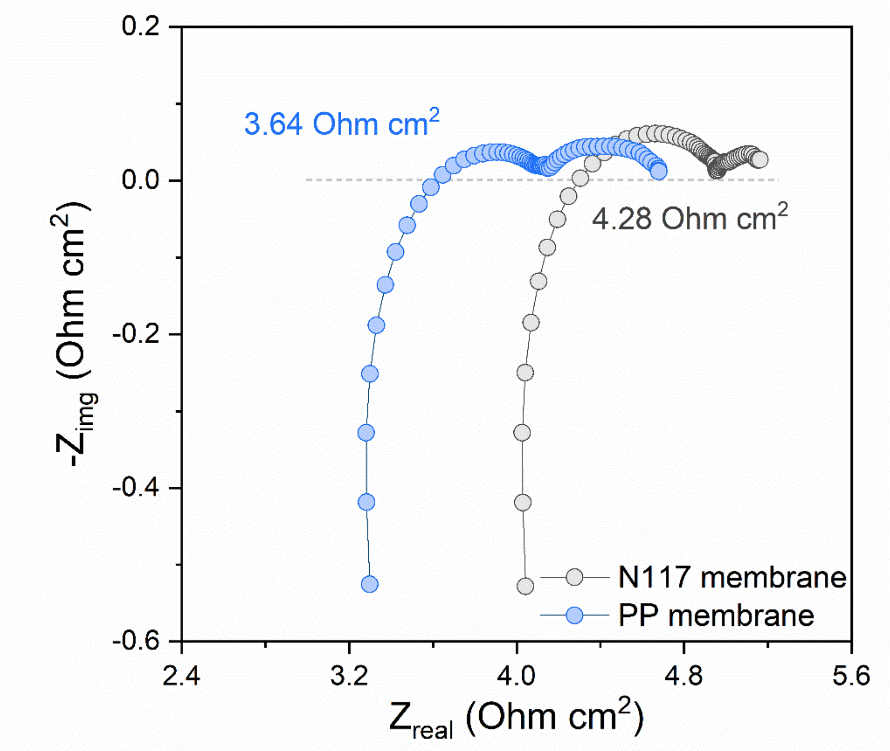

Supplementary Fig. 27 | The resistance test by alternating current mode. EIS of the Zn-I FBs flow cell using different membranes (PP with 1 M starch & N117 without starch) under charging to 50% SOC.

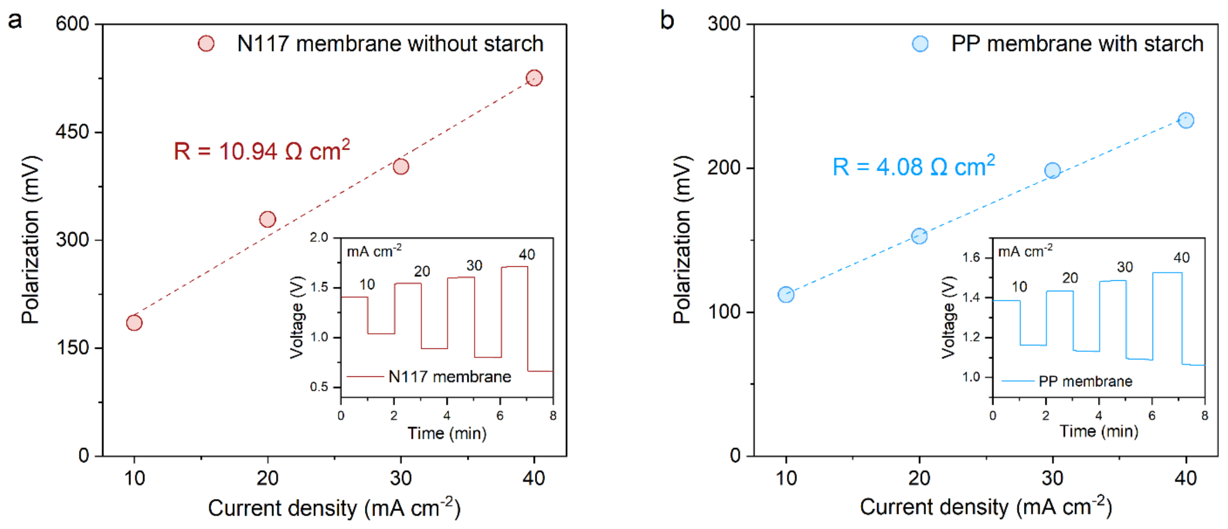

Supplementary Fig. 28 | The internal resistance tested by direct current mode. The relationship of polarization and current of the Zn-I FBs flow cell using different membranes (**a** PP membranes with 1 M starch & **b** N117 membranes without starch) under charging to 50% SOC at different current density (10 - 40 mA cm^{-2}) (The inset: polarization voltage under different current density). Note that the EIS results (**Supplementary Fig. 27**) can present the impedance measured under alternating currents (AC) within a certain AC frequency range, where the obtained resistance could not directly show the impedance of the battery in actual working conditions under direct currents (DC). For the batteries of internal resistance measured by DC mode, it includes all the resistance in the battery under realistic operation. To calculate the internal resistance of the FB during charging/discharging operation, we measured the overpotentials (U_2-U_1) when applying a specific magnification current (I). Thus, the resistance could be calculated as R= (U_2-U_1)/2I. It should be noted that the charge and discharge operation here lasted for a short period of time (1 min) to keep the 50% state of charge state of the polyiodide catholyte. Specifically, as shown in **Supplementary Fig. 28**, the calculated internal resistance of Zn-IS FBs with starch using a PP membrane was 4.08 Ω cm^2, which was lower than Zn-I FBs without starch using the N117 membrane (10.94 Ω cm^2). Thus, the difference (6.86 Ω cm^2) in internal resistance measured at 50% SOC of these two batteries could cause the difference as 9.65 mW cm^2 in power density at 37.5 mA cm^{-2}, almost aligned to their difference (13.17 mW cm^2 at 37.5 mA cm^{-2}, calculated by **Fig. 3c** in the manuscript) in the power density of the whole discharge process. Notably, a small gap value in the difference of power density between resistance-based calculation and power density test-based calculation would be caused by the concentration polarization and activation polarization in the overall FBs system.

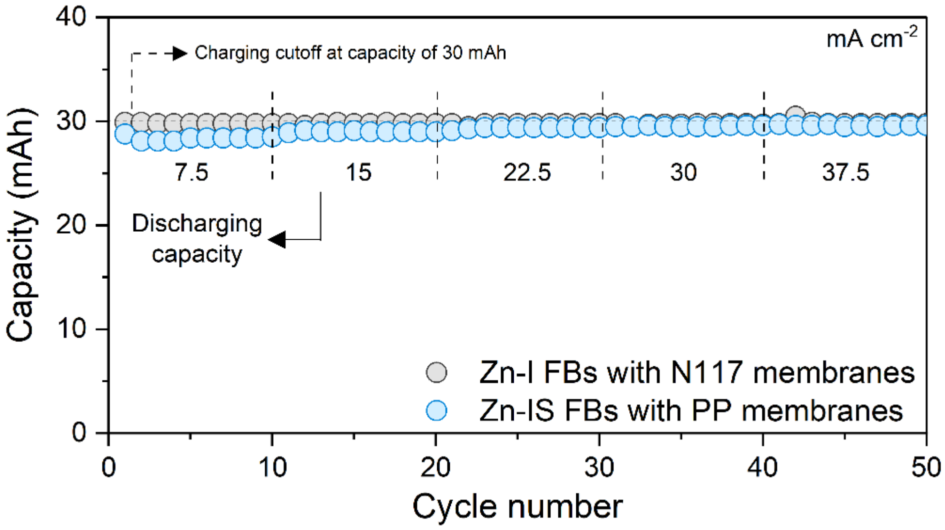

Supplementary Fig. 29 | The capacity retention using different membranes. Discharging capacity of the Zn-I FBs flow cell using different membranes (PP with 1 M starch & N117 without starch) during charging to 30 mAh at corresponding rate tests.

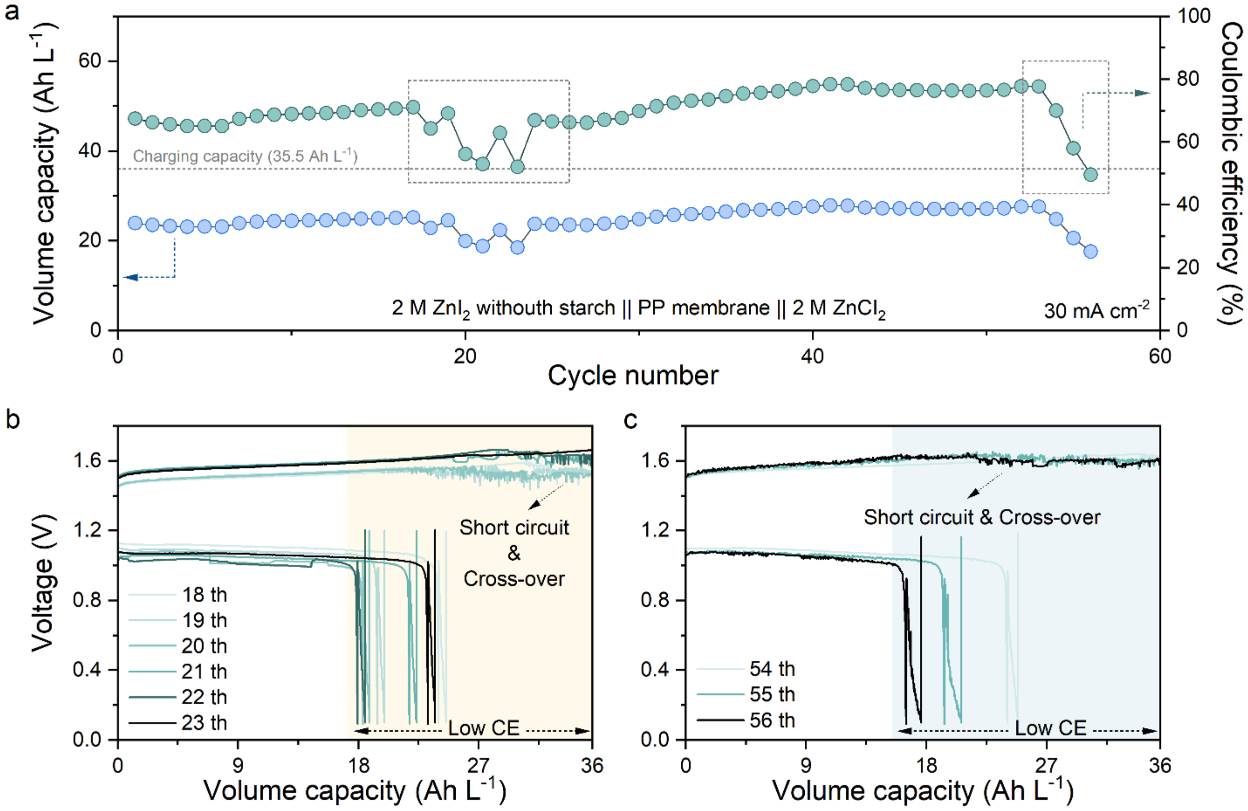

Supplementary Fig. 30 | long cycling of Zn-I FBs using PP membrane without starch. a Cycling performances of Zn-I FBs flow-cell system using PP membrane without starch at high volume capacity (33.5 Ah L^{-1}) under 22.5 mA cm^{-2}. Selected cycles corresponding to **b** region (18 th -23 th) and **c** region (54 th - 56 th) in **c**, where the short-circuit point and cross-over are marked.

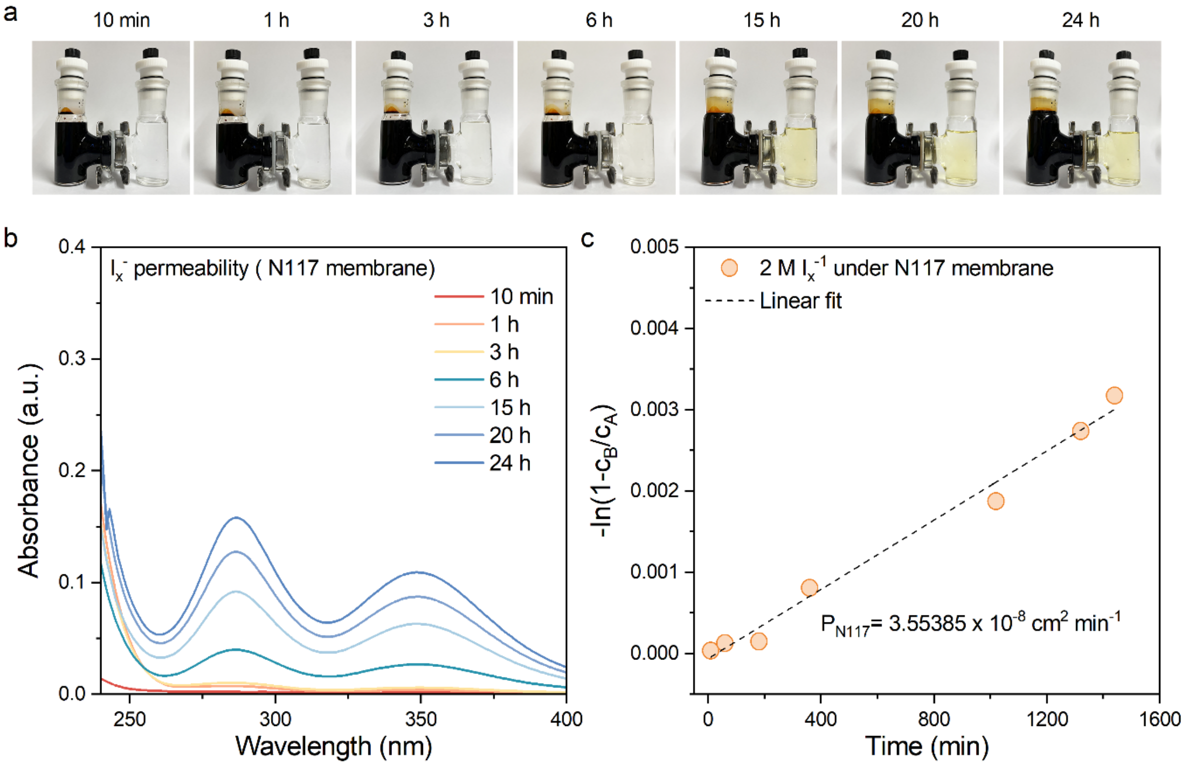

Supplementary Fig. 31 | KI_3 permeation measurements with N117 membrane. a Photographs of the KI_3 permeate solutions through N117 membranes under blank 2 M KI_3 electrolytes. **b** UV-vis of the KI_3 permeated side. **c** $-ln(1-c_B/c_A)$ *vs*. permeation time for the determination of permeability of KI_3 through N117 membranes under blank 2 M KI_3. The fits in the $-ln(1-c_B/c_A)$ vs. t (time) plots in **c** were obtained by linear fitting. As displayed in **Supplementary Fig. 31**, N117 membranes showed a low iodine permeability (P_{N117}= 3.55385 × 10^{-8} cm^2 min^{-1}). Although the migration of negatively charged iodine species is supposed to be rejected by the Donnan exclusion effect of the N117 membrane, the cross-over would still diffuse through the micropore and swelling channel of the N117 membrane.

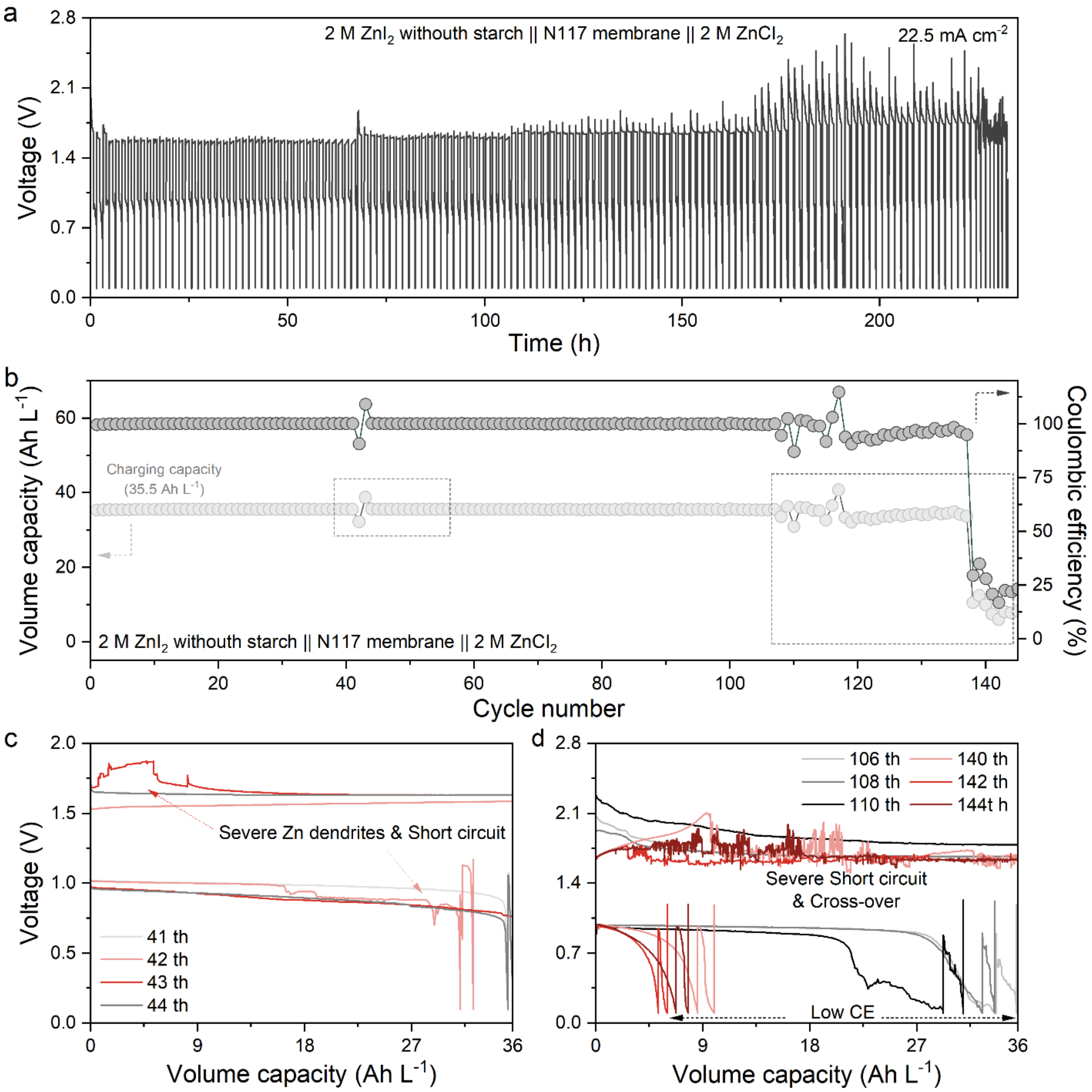

Supplementary Fig. 32 | long cycling of Zn-I FBs with N117 membranes. a Galvanostatic cycling of Zn-I FBs (2 ml of 2 M ZnI_2 without starch || N117 membrane || 8 ml of 2 M $ZnCl_2$, 4 cm^2 membrane area) at high volume capacity (33.5 Ah L^{-1}) under 22.5 mA cm^{-2}. **b** Cycling performances of Zn-I FBs flow-cell system using PP membrane without starch at high volume capacity (33.5 Ah L^{-1}) under 22.5 mA cm^{-2}. Selected cycles corresponding to **c** region (41 th - 44 th) and **d** region (106 th - 144 th) in **b**, where the soft short-circuit, severe short-circuit points and cross-over are marked.

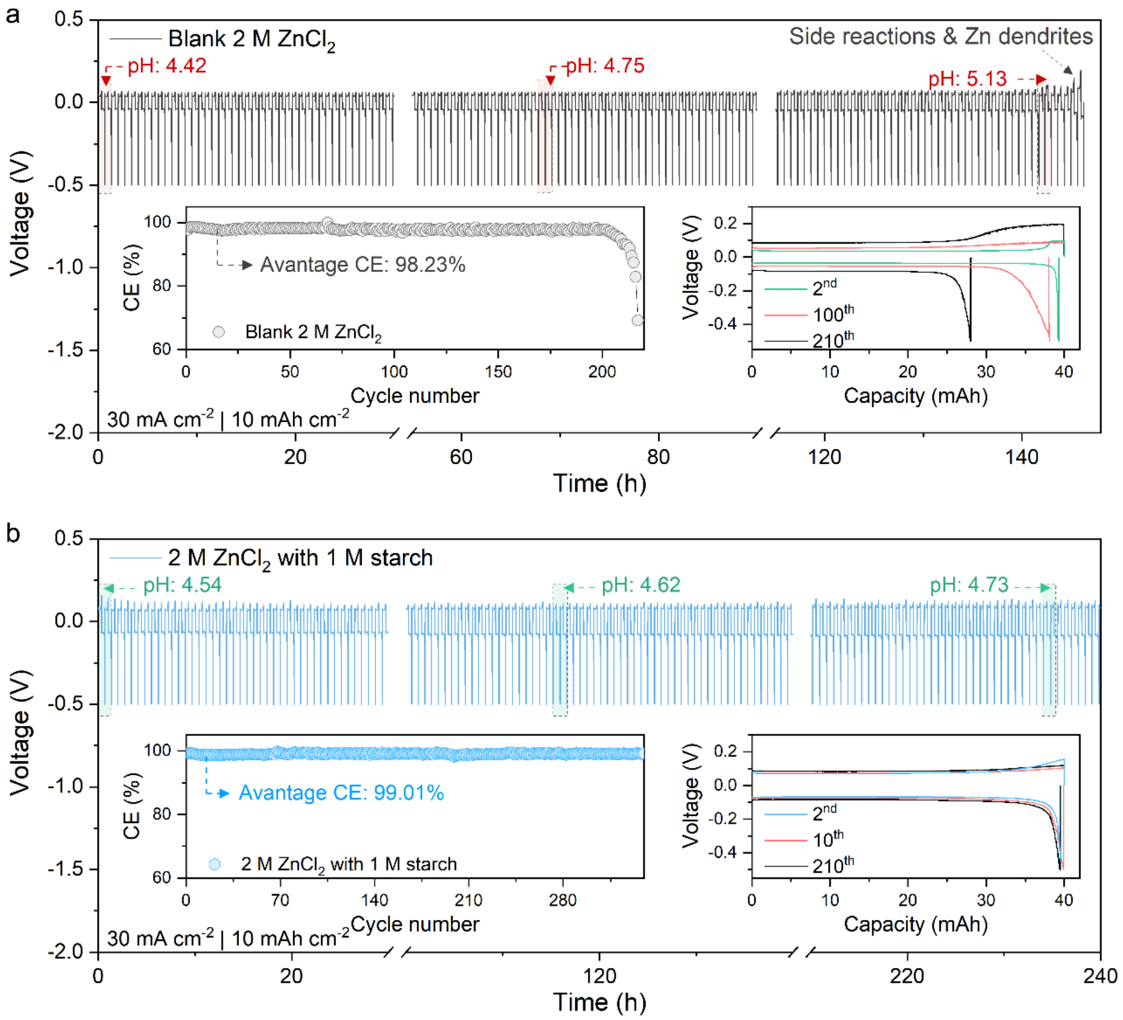

Supplementary Fig. 33 | Reversibility of Zn anode behaviors. Long cycling of Zn-based asymmetrical FBs (Zn foil@Carbon felt || PP membranes || Carbon felt) with **a** blank 2 M $ZnCl_2$ and **b** 2 M $ZnCl_2$ with colloidal 1 M starch additives at 10 mAh cm^{-2} under 30 mA cm^{2}. The insets show the representative CE, charging-discharging polarization curves and pH.

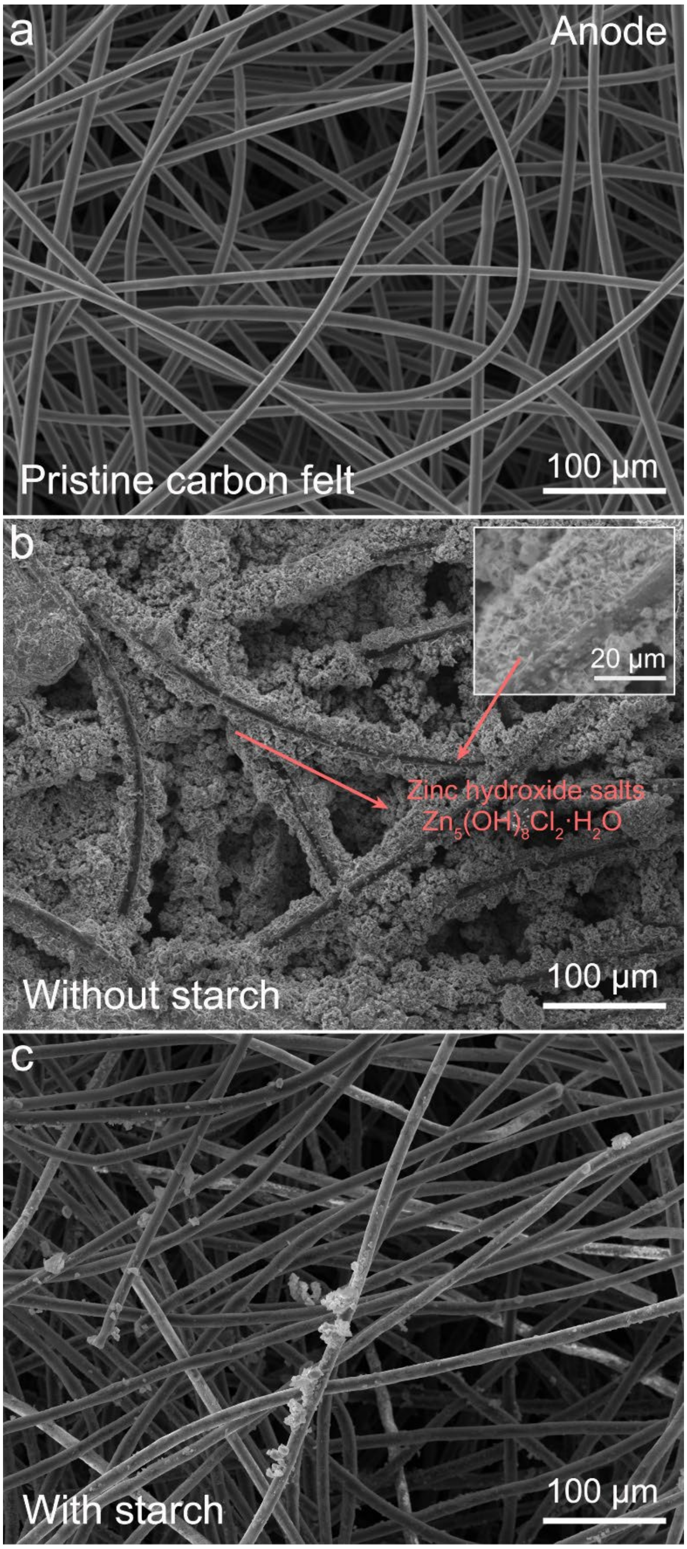

Supplementary Fig. 34 | Characterization of the electrode. SEM images of **a** pristine CF, **b** CF anode using PP membrane without starch, and **c** CF anode using PP membrane with starch in the discharging state at 22.5 mA cm^{-2} and 33.5 Ah L^{-1} after 30 cycles. (The inset **b**: the detail of the relevant electrode)

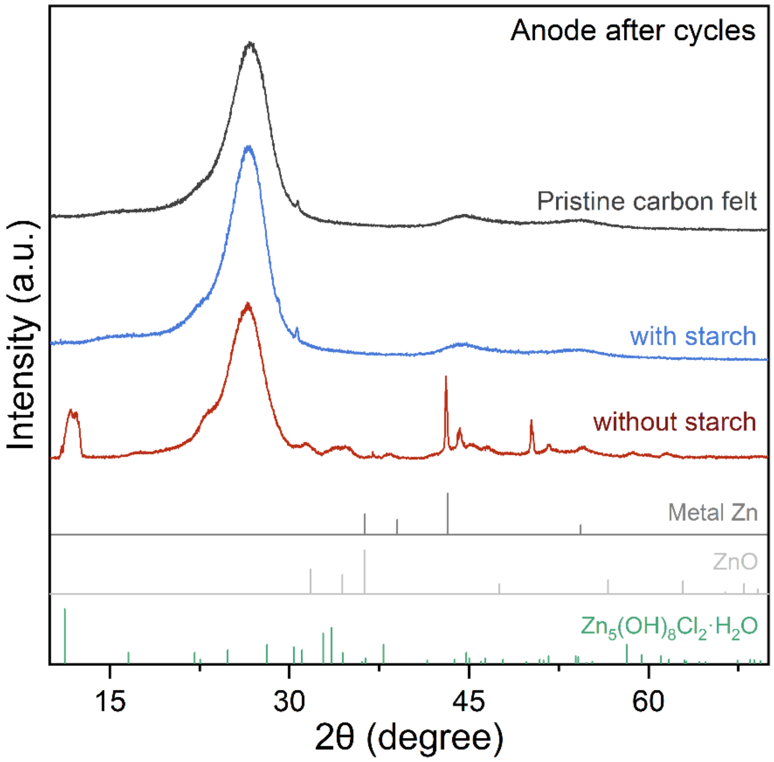

Supplementary Fig. 35 | Characterization of the electrode after cycles. XRD pattern of pristine CF, CF using PP membrane without starch, and CF using PP membrane with starch on the anodic side in the discharging state at 22.5 mA cm^{-2} and 33.5 Ah L^{-1} after 30 cycles, wherein PDF card of $Zn_5(OH)_8Cl_2{\cdot}H_2O$ and ZnO by-products are #07-0155 and #36-1451.[5,6]

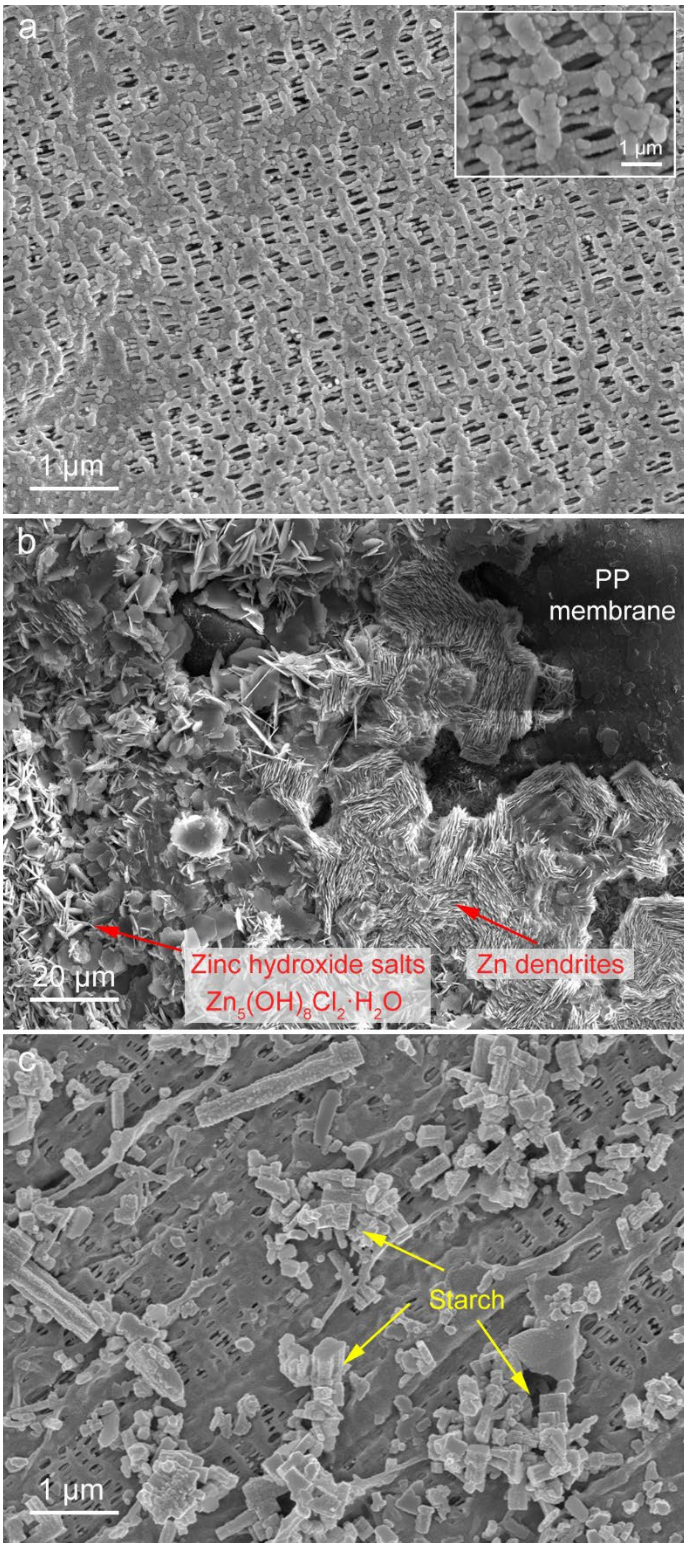

Supplementary Fig. 36 | Characterization of the cathodic electrode after cycles. SEM images of **a** pristine PP membrane, **b** PP membrane without starch and **c** with starch in the discharging state at 22.5 mA cm^{-2} and 33.5 Ah L^{-1} after 30 cycles. (The inset **a**: the detail of the relevant electrode)

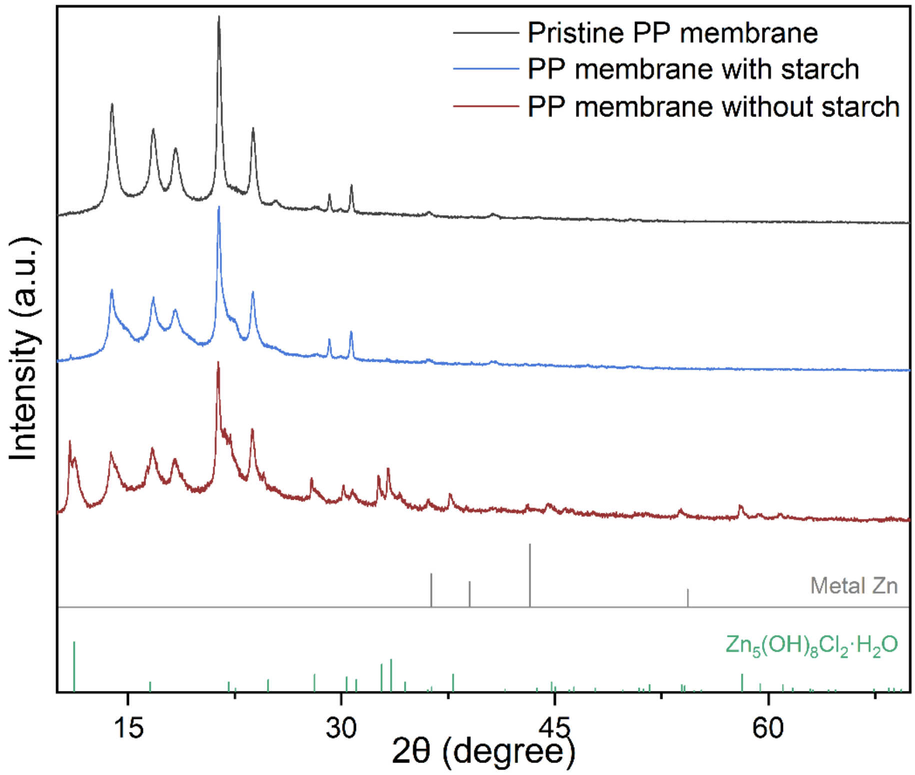

Supplementary Fig. 37 | Characterization of membranes after cycles. XRD pattern of pristine PP membrane, PP membrane with/without starch after cycles in the discharging state at 22.5 mA cm^{-2} and 33.5 Ah L^{-1} after 30 cycles, wherein PDF card of $Zn_5(OH)_8Cl_2 \cdot H_2$ is #07-015.[5,6]

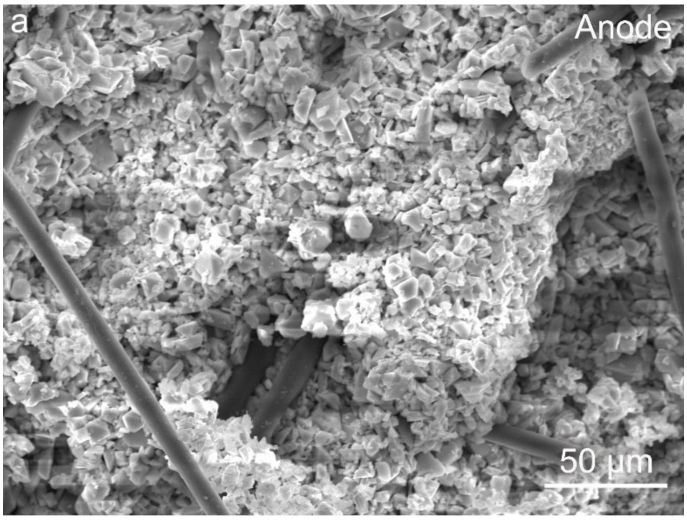

Supplementary Fig. 38 | Characterization of the electrode after cycles based on N117 membranes. SEM images of CF anode using N117 membrane without starch in the discharging state at 22.5 mA cm^{-2} and 33.5 Ah L^{-1} after 30 cycles.

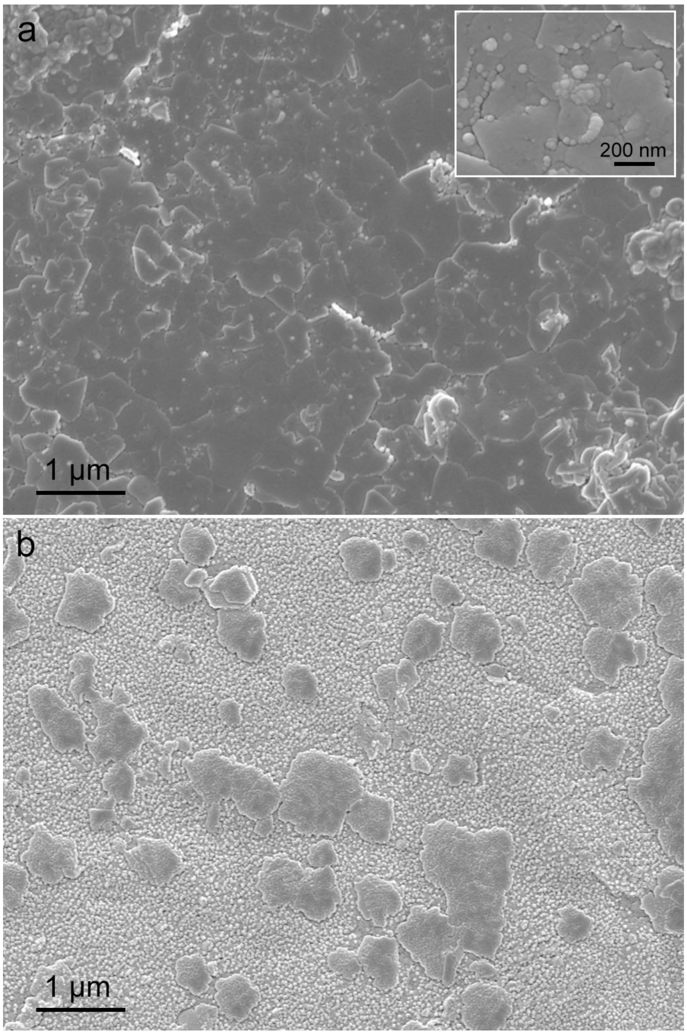

Supplementary Fig. 39 | Characterization of N117 membranes before and after cycles. SEM images of **a** pristine N117 membrane and **b** N117 membrane without starch on the anode side in the discharging state at 22.5 mA cm^{-2} and 33.5 Ah L^{-1} after 30 cycles. (The inset **a**: the detail of the relevant electrode).

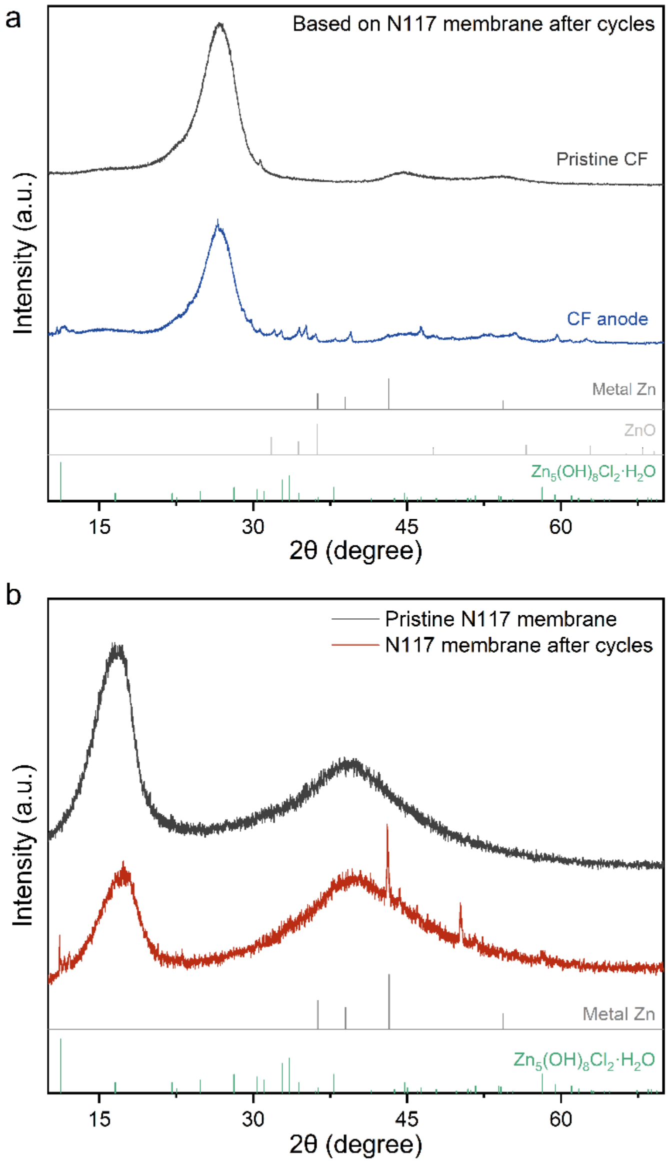

Supplementary Fig. 40 | Characterization of the electrode and membranes before and after cycles based on N117 membranes. XRD pattern of **a** CF anode using N117 membrane without starch and **b** N117 membrane without starch on the anode side in the discharging state at 22.5 mA cm^{-2} and 33.5 Ah L^{-1} after 30 cycles, wherein PDF card of $Zn_5(OH)_8Cl_2{\cdot}H_2O$ and ZnO by-products are #07-0155 and #36-1451.[5,6]

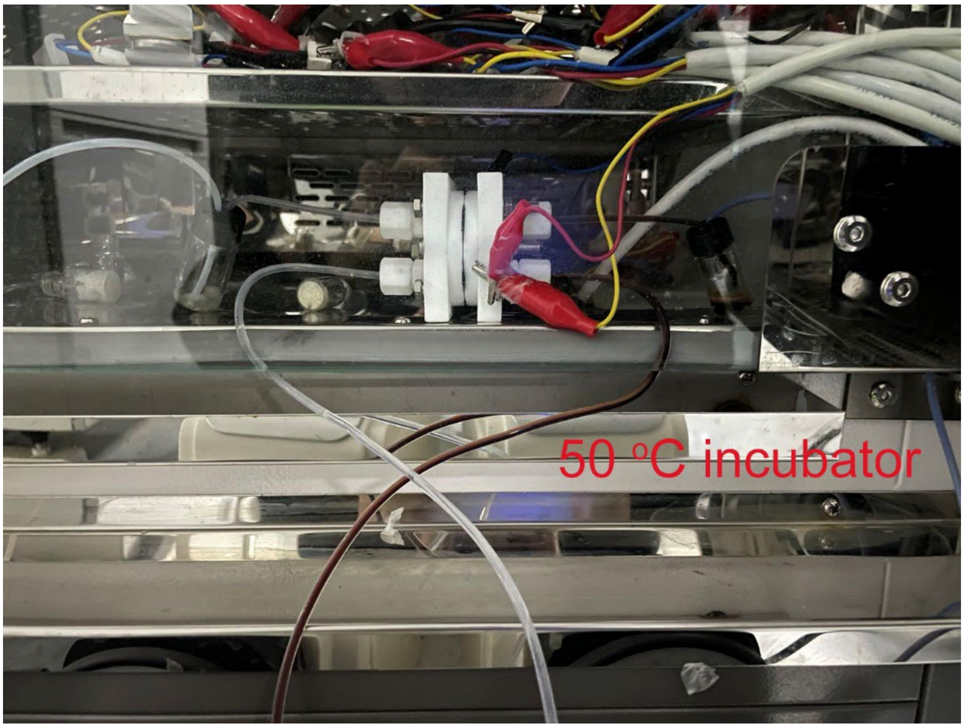

Supplementary Fig. 41 | High temperature conditions. Photographs of the Zn-IS FBs working in an incubator at a high temperature (50 °C).

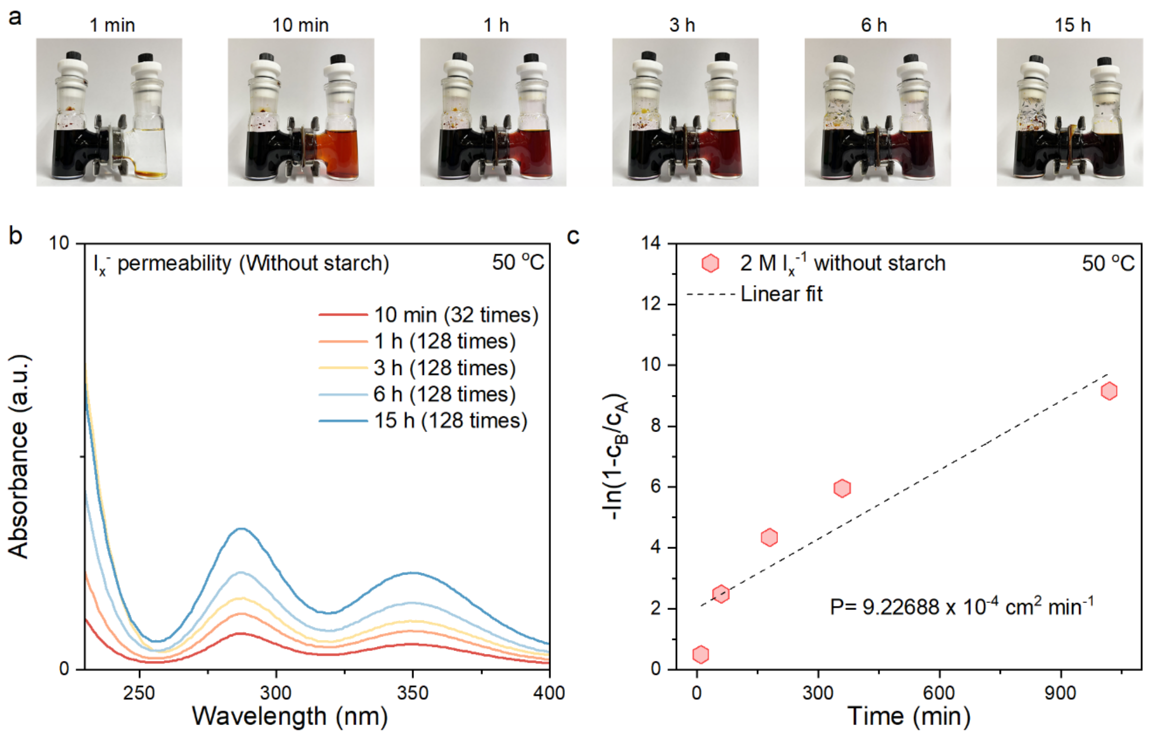

Supplementary Fig. 42 | KI_3 permeation measurements without starch under high temperature conditions. a Photographs of the KI_3 permeate solutions through PP membranes under blank 2 M KI_3 electrolytes at high temperature of 50 °C. **b** UV-vis of the KI_3 permeated side at high temperature of 50 °C. **c** $-ln(1-c_B/c_A)$ *vs.* permeation time for the determination of permeability of KI_3 through PP membranes under blank 2 M KI_3 at high temperature of 50 °C. The fits in the $-ln(1-c_B/c_A)$ vs. t (time) plots in **c** were obtained by linear fitting.

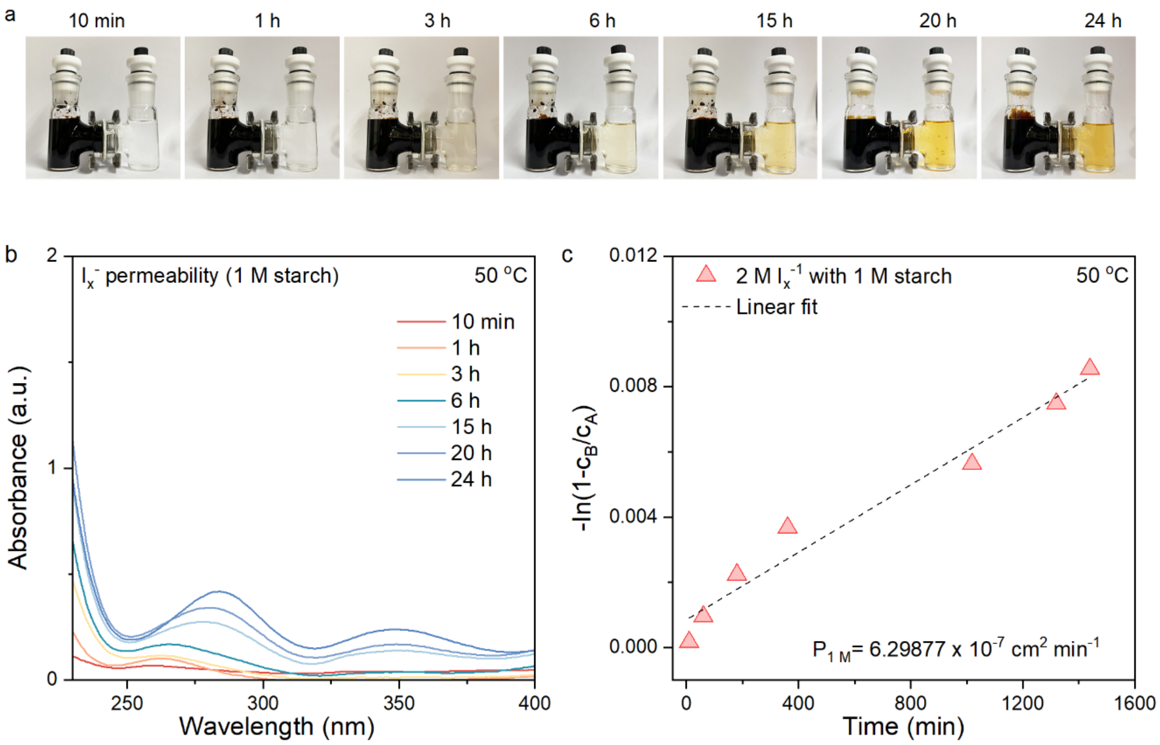

Supplementary Fig. 43 | KI_3 permeation measurements with starch under high temperature conditions. **a** Photographs of the KI_x permeate solutions through PP membranes under 2 M KI_x with 1 M starch electrolytes at high temperature of 50 °C. **b** UV-vis of the KI_x permeated side at high temperature of 50 °C. **c** $-\ln(1-c_B/c_A)$ *vs*. permeation time for the determination of permeability of KI_x through PP membranes under 2 M KI_x with 1 M starch at high temperature of 50 °C. The fits in the $-\ln(1-c_B/c_A)$ vs. t (time) plots in **c** were obtained by linear fitting.

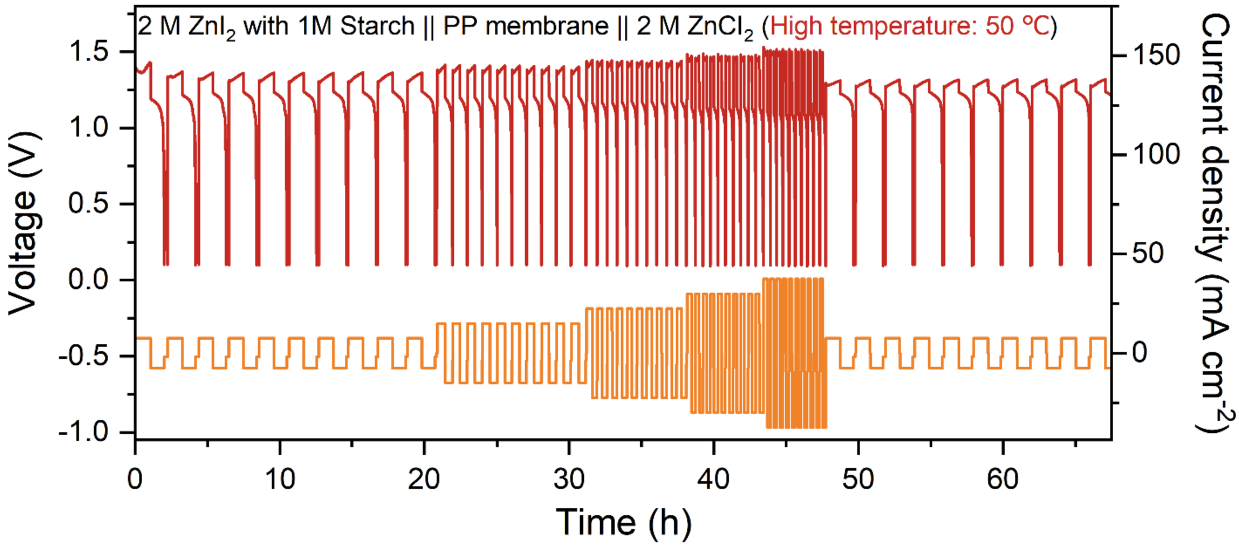

Supplementary Fig. 44 | Rate performance of Zn-IS FBs under high temperature conditions. Galvanostatic cycling of Zn-IS FBs (2 ml of 2 M ZnI_2 with 1 M starch || PP membrane || 8 ml of 2 M $ZnCl_2$, 4 cm^2 membrane area) under 7.5, 15, 22.5, 25, 30 and 7.5 mA cm^2 at high temperature (50 °C).

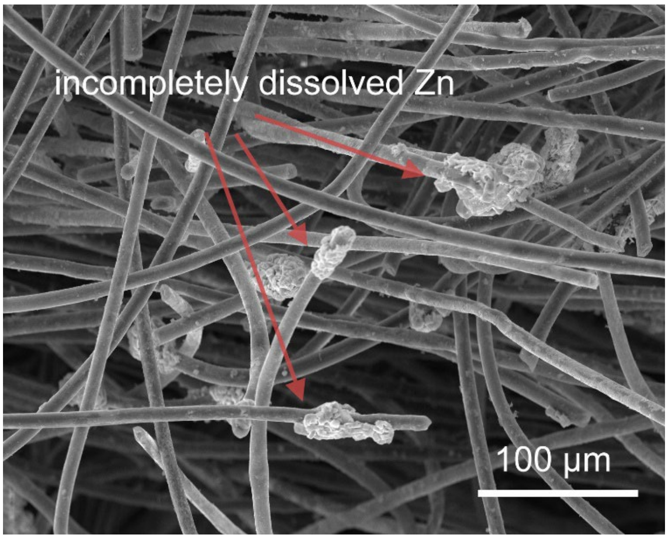

Supplementary Fig. 45 | The cathodic electrode after cycles. SEM images of CF anode using PP membrane with starch in the discharging state after 30 cycles at 50 ^{o}C condition.

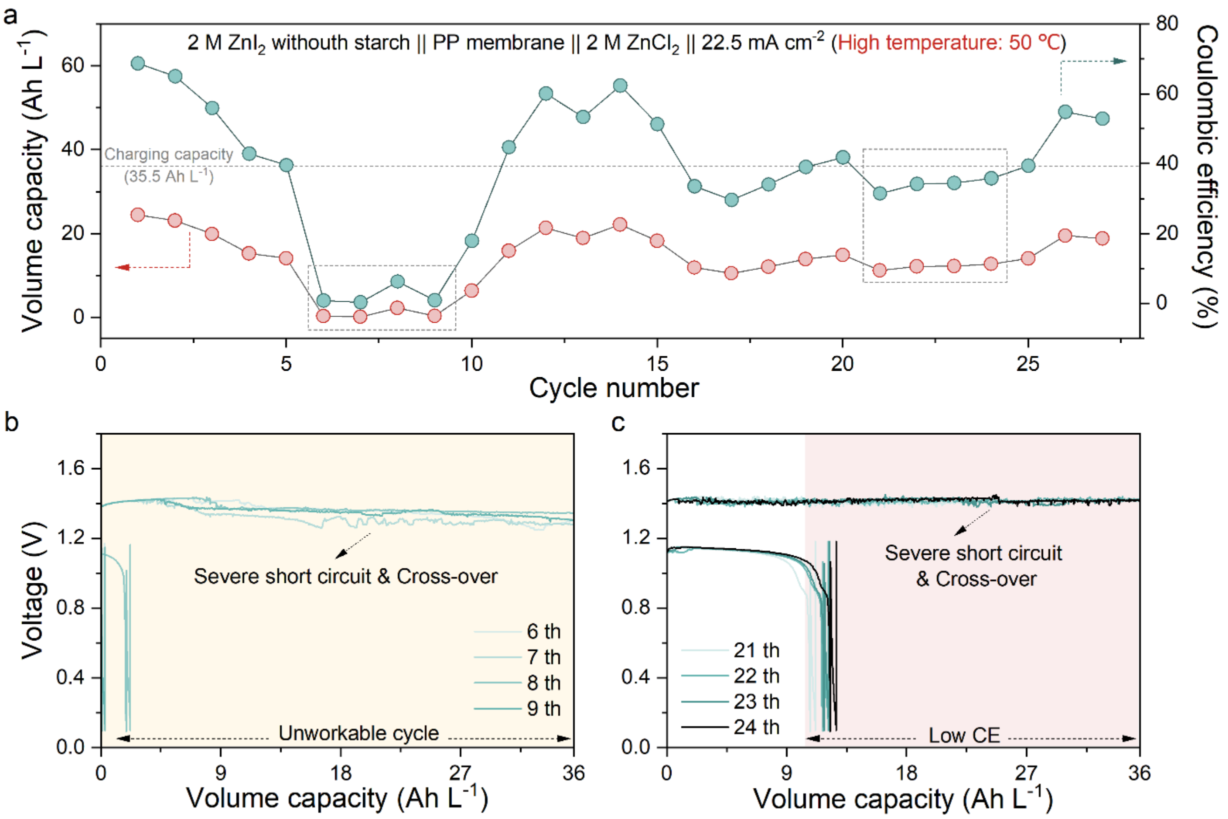

Supplementary Fig. 46 | long cycling of Zn-I FBs under high temperature conditions. a Cycling performances of Zn-I FBs flow-cell system using PP membrane without starch at high volume capacity (33.5 Ah L^{-1}) under 22.5 mA cm^{-2} and high temperature (50 °C). Selected cycles corresponding to **b** region (6 th - 9 th) and **c** region (21 th - 24 th) in **a**, where the short-circuit point and cross-over are marked.

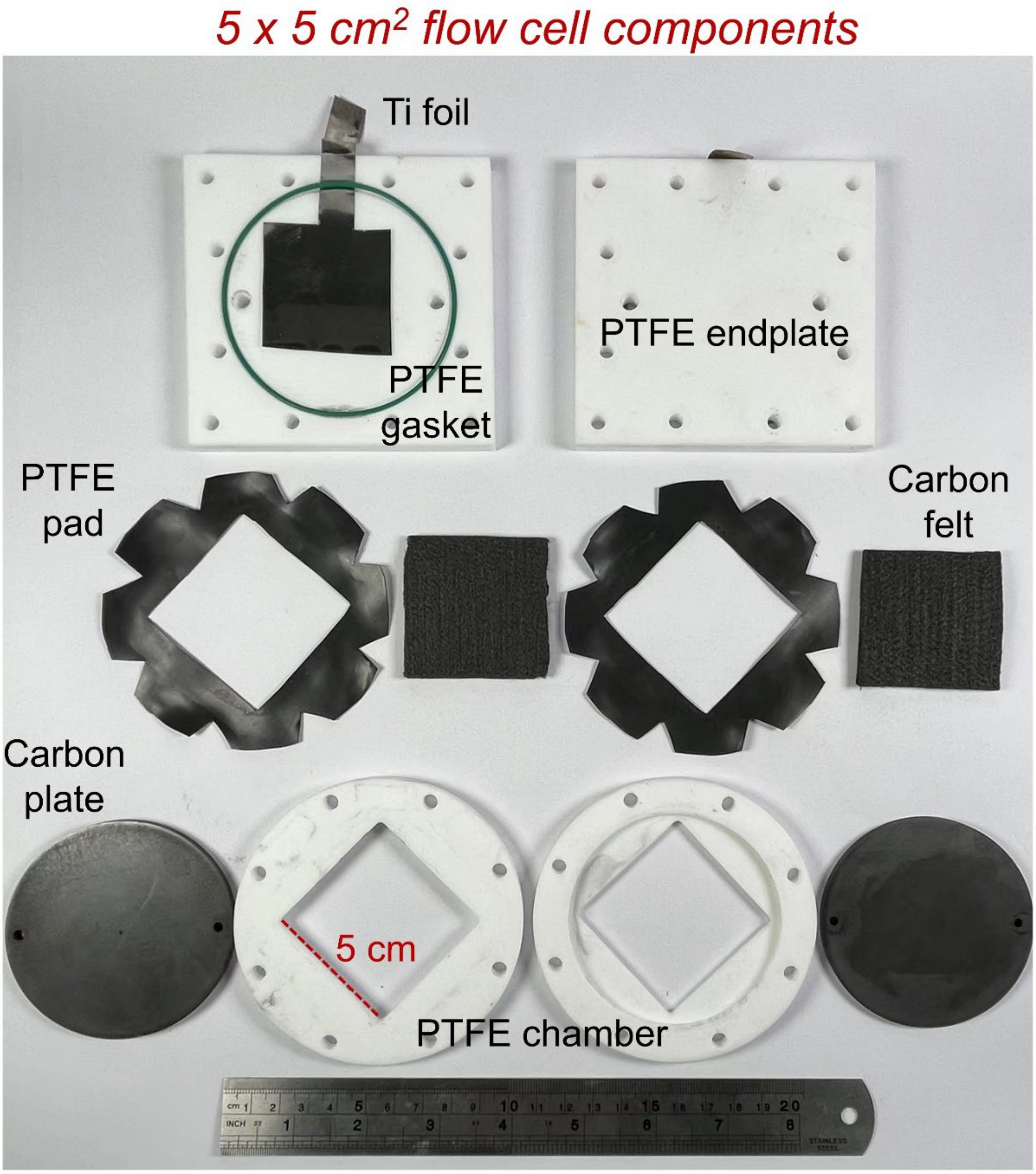

Supplementary Fig. 47 | Digital graphs of large-scale flow mode. Photographs of the cell components of 5×5 cm^2 cell for flow cell tests in **Fig.5**.

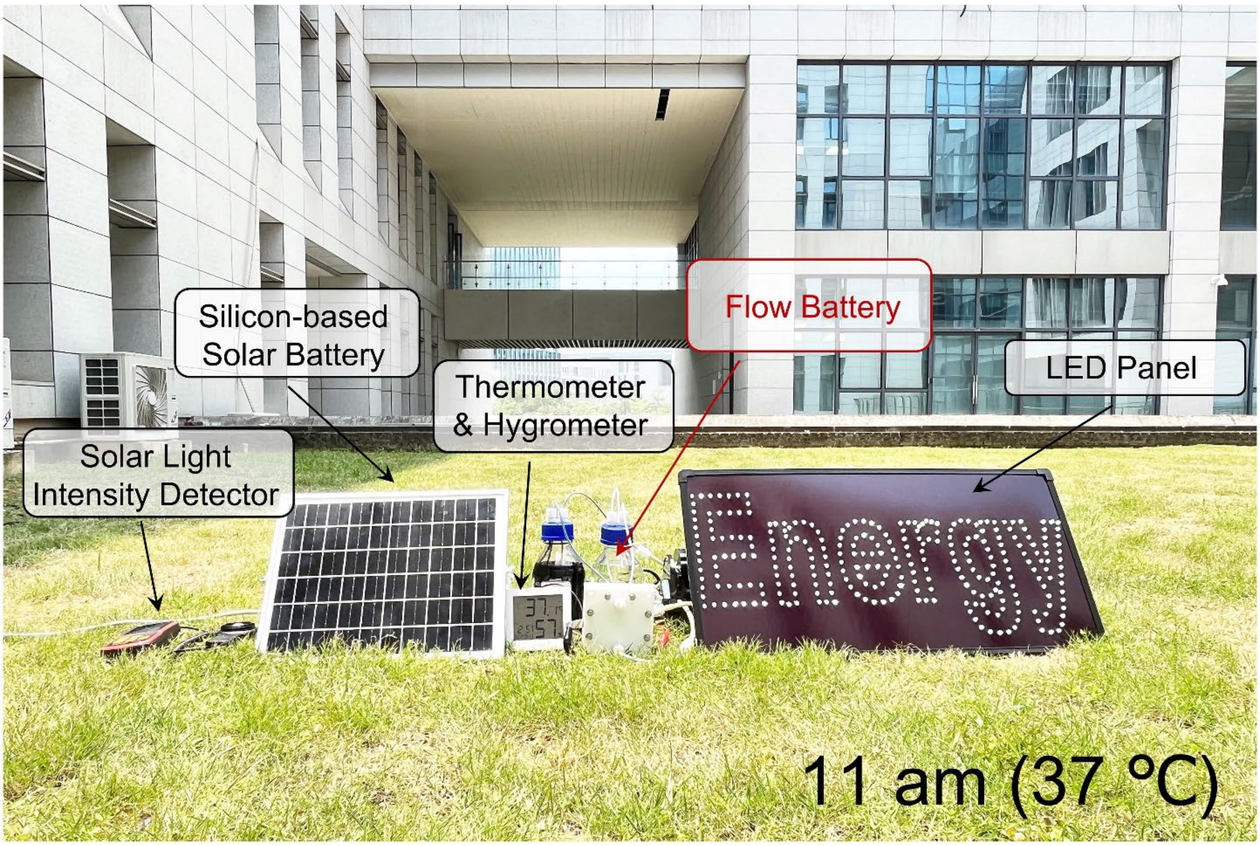

Supplementary Fig. 48 | Flow cell integrated with photovoltaic pack systems. Photographs of the components from the renewable solar-energy storage system.

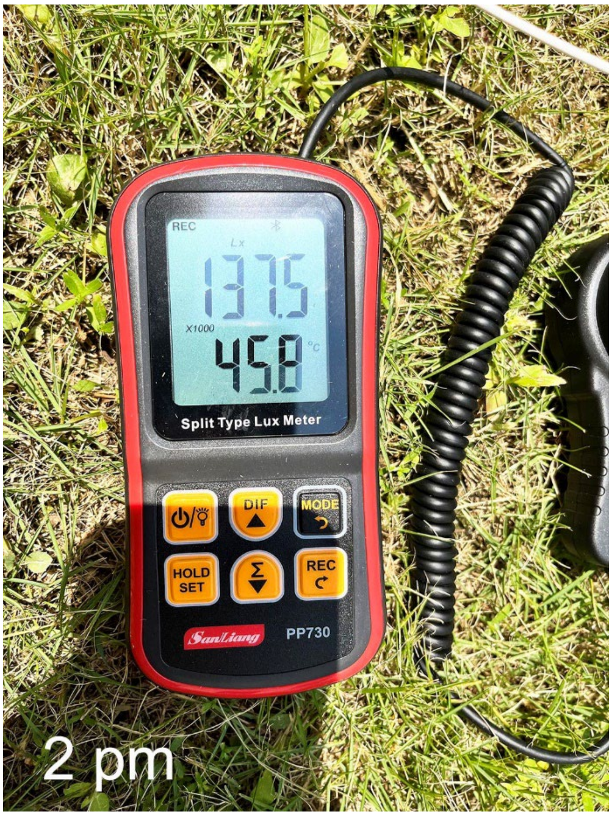

Supplementary Fig. 49 | Temperature measurement. The photograph of the measured ground temperature was about 45.8 °C at 2 pm.

Supplementary Table 1 | The components and their costs for a 1 MW Zn-I flow battery stack. According to the power density of long-term cycling at 22.5 mA cm^{-2} in **Fig. 3e**, the stack area of N117 membrane-based Zn-I FBs need about 5012 m^2 at 19.95 mW cm^{-2} for working 1 MW Zn-I FBs, and the stack area of PP membrane-based Zn-IS FBs only need about 3766 m^2 at 26.55 mW cm^{-2} for working 1 MW Zn-IS FBs. All component calculated from the average of the future state estimation at 2014.[7]

<table>
<tr><th>Component</th><th>Price per unit ($ m⁻²)</th><th>Amount of N117 membrane-based Zn-I FBs (m²)</th><th>Total cost of N117-based Zn-I FBs ($)</i></th><th><i>Amount of PP membrane-based Zn-I FBs (m²)</i></th><th><i>Total cost of PP membrane -based Zn-I FBs ($)</th></tr>
<tr><td>Bipolar plates</td><td>30</td><td rowspan="5">5012</td><td>150,360.00</td><td rowspan="5">3766</td><td>112,980.00</td></tr>
<tr><td rowspan="2">Membrane</td><td>PP: 10</td><td rowspan="2">2,506,000.00</td><td rowspan="2">37,660.00</td></tr>
<tr><td>N117: 500</td></tr>
<tr><td>Frame</td><td>15</td><td>75,180.00</td><td>56,490.00</td></tr>
<tr><td>Carbon felt</td><td>20</td><td>100,240.00</td><td>75,320.00</td></tr>
<tr><td>Gasket-1</td><td>1</td><td>4 pieces</td><td>4</td><td>4 pieces</td><td>4</td></tr>
<tr><td>Gasket-2</td><td>2</td><td>4 pieces</td><td>8</td><td>4 pieces</td><td>8</td></tr>
<tr><td colspan="2">Total cost for 1 MW Zn-I FBs</td><td colspan="2">$2,831,792.00</td><td colspan="2">$282,462.00</td></tr>
</table>

Supplementary Table 2 | The costs for a 1 MW Zn-I flow battery stack with 1 MWh. Note that all chemicals obtained from Alibaba. Available at https://www.alibaba.com/ (Accessed: 27th November 2023)

Chemicals	***Molecular weight ($g\ mol^{-1}$)***	***Price per unit ($\$\ kg^{-1}$)***	***Amount of chemicals (kg)***	***The cost of chemicals ($)***
$ZnCl_2$	136.28	11	6934.1	76,275.00
ZnI_2	319.22	17	16242.4	276,122.00
Starch	324.28	0.14	8249.9	1,154.00

Total cost for 1 MW N117 membrane-based Zn-I FBs with 1 MWh (stack + chemicals)	$3,184,189.00
Total cost for 1 MW PP membrane-based Zn-IS FBs with 1 MWh (stack + chemicals)	$636,013.00

Reference

1. Zhang, W. et al. Self-repairing interphase reconstructed in each cycle for highly reversible aqueous zinc batteries. *Nat. Commun.* **13**, 5348 (2022).
2. Lin, D. & Xing, B. Adsorption of phenolic compounds by carbon nanotubes: role of aromaticity and substitution of hydroxyl groups. *Environmental science & technology* **42**, 7254-7259 (2008).
3. Vaino, A. R. & Szarek, W. A. Iodine in carbohydrate chemistry. (2000).
4. Freedman, H. H. Intramolecular H-bonds. I. A spectroscopic study of the hydrogen bond between hydroxyl and nitrogen. *J. Am. Chem. Soc.* **83**, 2900-2905 (1961).
5. Xue, P. et al. A MOF-Derivative Decorated Hierarchical Porous Host Enabling Ultrahigh Rates and Superior Long-Term Cycling of Dendrite-Free Zn Metal Anodes. *Adv. Mater.* **34**, 2110047 (2022).
6. Zhang, M. Y. et al. Decavanadate doped polyaniline for aqueous zinc batteries. *Small* **18**, 2107689 (2022).
7. Darling, R. M., Gallagher, K. G., Kowalski, J. A., Ha, S. & Brushett, F. R. Pathways to low-cost electrochemical energy storage: a comparison of aqueous and nonaqueous flow batteries. *Energy Environ. Sci.* **7**, 3459-3477 (2014).
